# Characterization of the Cardiac Overexpression of *HSPB2* Reveals Mitochondrial and Myogenic Roles Supported by a Cardiac HspB2 Interactome

**DOI:** 10.1371/journal.pone.0133994

**Published:** 2015-10-14

**Authors:** Julianne H. Grose, Kelsey Langston, Xiaohui Wang, Shayne Squires, Soumyajit Banerjee Mustafi, Whitney Hayes, Jonathan Neubert, Susan K. Fischer, Matthew Fasano, Gina Moore Saunders, Qiang Dai, Elisabeth Christians, E. Douglas Lewandowski, Peipei Ping, Ivor J. Benjamin

**Affiliations:** 1 Microbiology and Molecular Biology Department, Brigham Young University, Provo, UT, 84602, United States of America; 2 Laboratory of Cardiac Disease, Redox Signaling and Cell Regeneration, Division of Cardiology, University of Utah School of Medicine, Salt Lake City, UT, 84132, United States of America; 3 Division of Cardiovascular Medicine, Dept. of Medicine, Medical College of Wisconsin, Milwaukee, WI, 53226, United States of America; 4 Program in Integrative Cardiac Metabolism, Center for Cardiovascular Research, University of Illinois at Chicago College of Medicine, Chicago, IL, 60612, United States of America; 5 UCLA Departments of Physiology, Medicine, and Cardiology, Los Angeles, CA, 90095, United States of America; UMCG, NETHERLANDS

## Abstract

Small Heat Shock Proteins (sHSPs) are molecular chaperones that transiently interact with other proteins, thereby assisting with quality control of proper protein folding and/or degradation. They are also recruited to protect cells from a variety of stresses in response to extreme heat, heavy metals, and oxidative-reductive stress. Although ten human sHSPs have been identified, their likely diverse biological functions remain an enigma in health and disease, and much less is known about non-redundant roles in selective cells and tissues. Herein, we set out to comprehensively characterize the cardiac-restricted Heat Shock Protein B-2 (HspB2), which exhibited ischemic cardioprotection in transgenic overexpressing mice including reduced infarct size and maintenance of ATP levels. Global yeast two-hybrid analysis using HspB2 (bait) and a human cardiac library (prey) coupled with co-immunoprecipitation studies for mitochondrial target validation revealed the first HspB2 “cardiac interactome” to contain many myofibril and mitochondrial-binding partners consistent with the overexpression phenotype. This interactome has been submitted to the Biological General Repository for Interaction Datasets (BioGRID). A related sHSP chaperone HspB5 had only partially overlapping binding partners, supporting specificity of the interactome as well as non-redundant roles reported for these sHSPs. Evidence that the cardiac yeast two-hybrid HspB2 interactome targets resident mitochondrial client proteins is consistent with the role of HspB2 in maintaining ATP levels and suggests new chaperone-dependent functions for metabolic homeostasis. One of the HspB2 targets, glyceraldehyde 3-phosphate dehydrogenase (GAPDH), has reported roles in HspB2 associated phenotypes including cardiac ATP production, mitochondrial function, and apoptosis, and was validated as a potential client protein of HspB2 through chaperone assays. From the clientele and phenotypes identified herein, it is tempting to speculate that small molecule activators of HspB2 might be deployed to mitigate mitochondrial related diseases such as cardiomyopathy and neurodegenerative disease.

## Introduction

Studies of molecular chaperones and their regulatory pathways are among the most challenging and illuminating ones for understanding the hierarchical integration among genotype-phenotype relationships, biological systems and cellular networks [1–3]. Among their multifaceted functions, molecular chaperones transiently interact with other proteins to facilitate protein folding, translocation, and degradation, all of which contribute to the quality control requirements for maintaining homeostasis of the proteome (termed “proteostasis”). In parallel, systems biology has emerged as a powerful organizing principle for integrating biophysical properties, creating biological functions of complex webs of macromolecular interactions (termed the “interactome” network). How molecular (HSP) chaperones might be inextricably linked to interactome networks and interrelated organelles in cardiac health and disease remain poorly defined.

In recent decades, attempts to understand genotype-phenotype relationships have been significantly aided by the characterization of inheritable Mendelian traits and experimental genetic maneuvers such as transgenesis and gene targeting in model organisms. In the present context, owing to the head-to-tail genomic organization of the genes encoding HspB2 (also known as Myotonic Dystrophy Protein Kinase Binding Protein, MKBP [4]) and HspB5 (also known as alpha-B crystallin, CryAB), Brady and collaborators had originally set out to create the single *HSPB5* knockout but inadvertently eliminated both genes resulting in double knockout (DKO) mice [5]. Because DKO mice survived into adulthood, these initial studies were the first to illustrate that both *HSPB2* and *HSPB5* deficiency was dispensable for early and postnatal development with relative mild effects of cardiac hypertrophy (~10% heart weight) in adult hearts, perhaps due to redundancy among sHSPs [5, 6].

Further characterization of DKO hearts by Morrison et al. (2004) revealed severely diminished levels of total reduced glutathione (GSH, 56%) and increased oxidized glutathione (GSSG) when compared with wild type (WT) mice, suggesting that DKO hearts are under higher levels of oxidative stress. Evidence of sHSPs in redox state regulation is based on our earlier work that showed that knockout of heat shock transcription factor 1 (HSF1) in mice not only lowered basal levels of HspB5 expression but also decreased GSH content [7]. Whereas *HSPB5* deficiency could contribute to the lower GSH in the DKO mice, these findings could not exclude a role for HspB2, which not only interacts with the outer membrane of mitochondria [8] but has been hypothesized to regulate mitochondria energetics [9]. Taken together, these studies support the general notion that genotype-phenotype relationships are governed by higher order complexities, which, in part, are related to variable expressivity, genetic modifiers, incomplete penetrance, redundancy, and age-related conditions.

In an attempt to unmask tissue-specific roles of HspB2, which can be hidden by overlapping sHSP function, we report here on the phenotypes of cardiac *HSPB2* overexpression (OE) as well as a large-scale cardiac HspB2 protein interactome. HspB2 OE mice (HSPB2cTg) protected cells from ischemia/reperfusion (I/R) and enhanced mitochondrial recovery with improved ATP levels at reperfusion. These results were supported by a cardiac protein interactome for HspB2 achieved through Y2H and co-immunoprecipitation (co-IP) approaches. A combined total of 149 HspB2 binding partners are reported, many of which are mitochondrial. This interacome showed specificity for HspB2 in that HspB5 binds only a subset of HspB2 binding partners. The HspB2 interactome was further supported by preliminary validation of an HspB2 target, glyceraldehyde 3-phosphate dehydrogenase (GAPDH) as an *in vivo* client. Many of these newly discovered HspB2 binding partners are linked to myopathies or neurodegenerative disease, suggesting a role for HspB2 in these debilitating human conditions.

## Materials and Methods

### HSPB2cTg mouse line and controls

**Fig 1:**
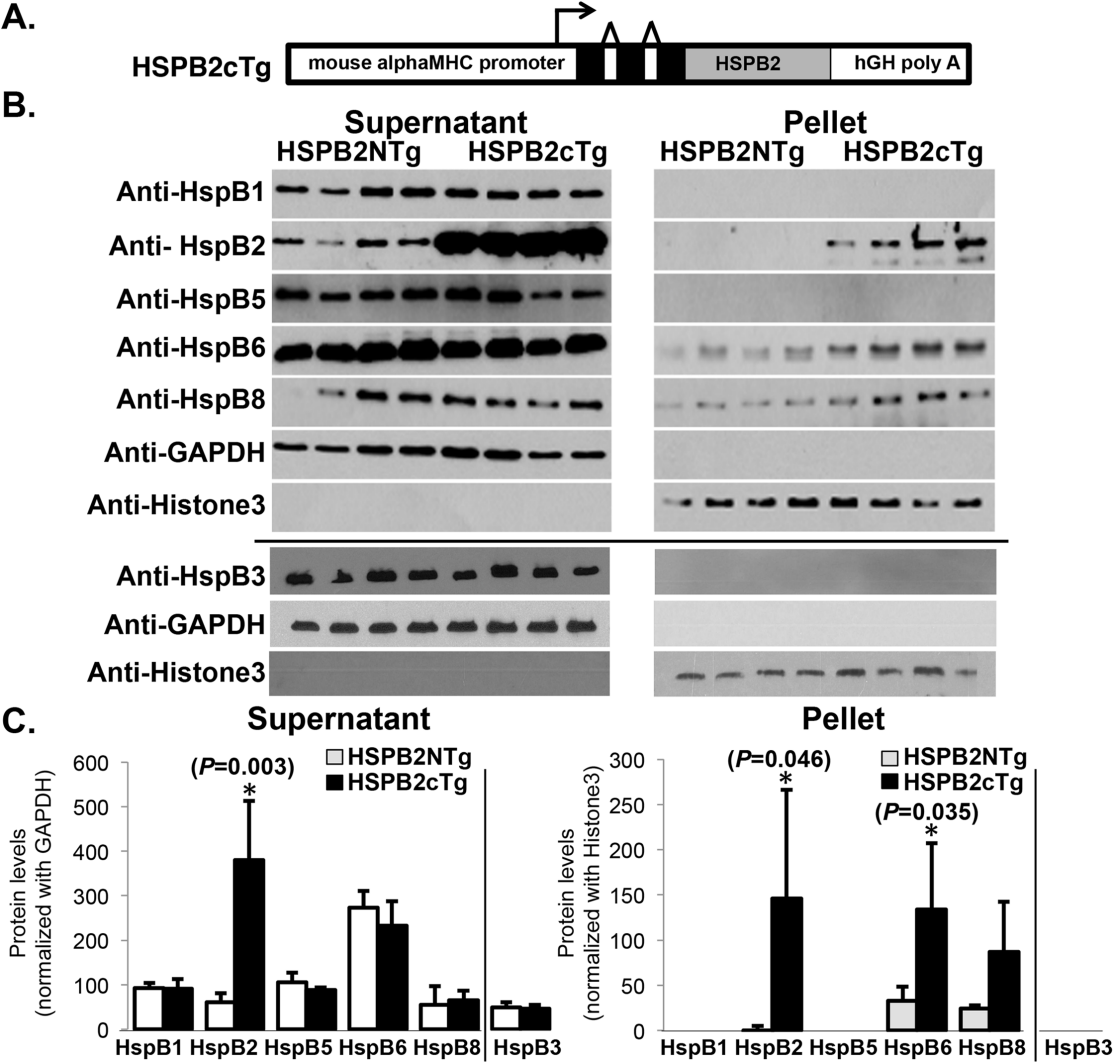
Generation of *HSPB2* cardiac specific overexpressors and effects on sHSP expression in transgenic mice (HSPB2cTg). **(A)** A schematic diagram illustrates the human heat shock protein B2 (HSPB2) under the control of the α-myosin heavy chain (α-MyHC) promoter: the 3 black boxes in the promoter represent noncoding exons in the 5’-untranslated region [10] and polyA splicing is provided by the human growth hormone sequences downstream of the inserted cDNA (hGH). **(B)** Representative Western blots of cardiac samples of transgenic (HSPB2cTg) mice and non-transgenic (HSPB2NTg) littermates (n = 4 animals per group) prepared as detergent soluble (supernatant) and non-soluble (pellet) portions, respectively. Protein levels of HSPB2 expression is markedly elevated (~4 x fold) in the HSPB2cTg hearts compared with non-transgenic (HSPB2NTg) littermates when assessed by western blot using anti-HspB2 antibodies [9]. Representative small heat shock proteins (sHSPs—HspB1, HspB3, HspB5, HspB6 and HspB8) exhibit similar levels in detergent soluble extracts, while HspB6 is significantly elevated in detergent insoluble fractions. Protein concentration was determined and western blots were performed using anti-sHSP (HspB1, HspB2, HspB5, HspB6 and HspB8) with anti-histone3 and anti-GAPDH as loading controls as previously described [9]. In addition, anti-HSPB3 antibodies were obtained from StressMarq Biosciences Inc and HspB3 levels were determined separately, which is indicated by a black line. **(C)** Densitometry-based quantification of the levels of protein in the immunoblots in Fig 1B. * *P*<0.05 when compared with corresponding HSPB2NTg group.

Human cDNA of *HSPB2* was amplified and inserted under the control of the α-myosin heavy chain (α-MHC) (Fig 1A) [10]. Transgenic [11] mice were generated by microinjection of fertilized embryos and maintained in a mixed genetic background of the C57Bl/6J and 129S6 strains. Genotyping of the HSPB2cTg mice was performed by PCR using the following primers: forward aggcagggaagtggtggtgtagg and reverse ggccttctccgaagcgctgc, respectively. Mice overexpressing cardiac *HSPB2* are referred to as HSPB2cTg and their corresponding Flox wild type control littermates as HSPB2NTg, whereas the cardiac *HSPB2* KO and littermate control are HSPB2cKO and HSPB2wt, respectively.

### Western blot analysis of heart tissue

Mouse hearts were removed and rinsed with ice cold PBS as previously described from our laboratory [9]. The tissue was minced into small pieces and homogenized in 0.25 ml of 50 mM Tris–HCl, pH 7.4, 150 mM NaCl, 1 mM EDTA, 1% Triton X-100. Following incubation on ice for 30 m, the lysates were clarified by centrifugation at 12,000 rpm for 10 m at 4°C. The supernatants were used as detergent-soluble fractions. The resulting pellet fractions were washed twice and sonicated in 100 μl 1× SDS buffer (50 mM Tris–HCl, pH 6.8, 2% SDS, 100 mM DTT, 10% Glycerol) for 30 s as detergent-insoluble fractions. Protein concentration was determined and western blots were performed using anti-sHSP (HspB1, HspB2, HspB5, HspB6 and HspB8) with anti-histone3 and anti-GAPDH as loading controls as previously described [9]. All antibodies were commercially available as described [9], with the exception of the HspB2 antibody which we purchased through 21^st^ Century Biochemicals using the following peptide sequence: CPATAEYEFANPSRLGEQ-amide. In addition, anti-HspB3 antibodies were obtained from StressMarq Biosciences Inc. and HspB3 levels were determined on a separate western blot.

### Ischemia reperfusion *in vivo*

Age-matched male mice (10–12 week-old) selected for the study were bred according to approved guidelines of Institutional Animal Care and Use Committee (IACUC) at the University of Utah. Animals were anesthetized with a ketamine (100mg/kg) and xylazine (0.5mg/kg) cocktail administered intraperitoneally. Following tracheal intubation of the trachea, the mouse was placed on a small rodent ventilator at a respiratory rate of 120 breaths per m and 0.25 ml tidal volume. To maintain temperature control, animals were placed on a water-heated surgical bed (E-Z Anesthesia, US State) with temperature setting at 37°C. Under sterile conditions, the heart was exposed through the 4^th^ intercostal space and a 7–0 suture, attached to a balloon, was applied around left descending coronary artery [12]. The cardiac ischemia was introduced by inflating the balloon while using continuous electrocardiographic (ECG) monitoring. Myocardial ischemia was monitored by the appearance of pale color of the lateral myocardial wall to the apex, S-T segment elevation, widening of the QRS, and/or ventricular arrhythmias on continuous ECG. The duration of ischemia lasted 45 m before the balloon was deflated and suture tie removed to initiate reperfusion. The chest cavity was closed before extubation of the animal.

### Assessment of infarction size

Twenty-four hours (h) after reperfusion start, the mice were euthanized, the hearts were excised and cannulated with a 20-gauge needle through the aorta, and then the hearts were perfused with Krebs-Henseleit solution [13]. The LAD was sutured and tied at the site of previous occlusion to permit perfusion of the aorta with 5% Evans Blue, which stains the non-risk area dark blue. The hearts were trimmed to leave only the left ventricle, which was frozen and sliced into 6–7 pieces. Staining in a 1% solution of 2,3,5-triphenyltetrazolium chloride (TTC) at 37°C for 30 m resulted in viable tissue and infarct tissue appearing in red and white colors, respectively. Each piece of heart was photographed (Dino-Lite) on both sides and images were processed with ImagePro Plus 7.0 software. The infarction mass of each piece was calculated as white (infarcted) average of both sides, divided by the sum of red plus white [14] average of both sides, then multiplied by the mass of that piece. Total infarction was calculated by the sum of the mass of infarction divided by the sum of the mass of risk as previously described from our laboratory [13].

### Troponin I measurement

Mouse blood samples were set for clotting before centrifugation for 15 m at 800g. The serum was collected, diluted in series from 1:10 to 1:1000, and then loaded into a 96-well sample plate from the High Sensitive Mouse Cardiac Troponin-I kit (Life Diagnostics, Inc.). Troponin I level was measured at 450 nm by following the manufacturers recommendations.

### Mitochondria swelling assay

Mitochondria were isolated from mouse hearts as previously described [9] and diluted to 250 ug/ml in mitochondria swelling buffer containing 120 mM KCl, 10 mM Tris, 5 mM KH_2_PO_4_. The mitochondria were equilibrated for 5 m at room temperature and 250 uM CaCl_2_ was loaded to induce swelling. Data was read at 540 nm.

### Bioenergetics consequences

For isolated heart studies and ^31^P NMR data collection, mice were heparizined (50 U/10 g, i.p.) and anesthetized (ketamine, 80 mg/kg, plus xylazine, 12 mg/kg, i.p.). Excised hearts were retrogradely perfused (60 mm Hg) with modified Krebs–Henseleit buffer (118.5 mM NaCl, 4.7 mM KCl, 1.5 mM CaCl_2_,1.2 mMMgSO_4_ and 1.2 mMKH_2_PO_4_) equilibrated with 95% O_2_/5% CO_2_, at 37°C, and containing 0.4 mM palmitate/fatty acid free albumin complex (3:1 molar ratio) and 10 mM glucose [15]. A water-filled latex balloon was placed into the left ventricle and set to a diastolic pressure of 5 mm Hg to provide a physiological load.

The isolated perfused hearts were placed inside a 10 mm broadband NMR radiofrequency probe within a 14.1 T NMR magnet (Bruker Biospin, Billerica, MA) for collection of sequential, ^31^P NMR spectra. ^31^P NMR data provided detection of high-energy phosphate content, including phosphocreatine (PCr) and ATP throughout an I/R protocol. Following collection of baseline, pre-ischemic baseline NMR data, perfused hearts were subjected to 16 m of global ischemia at 37°C, followed by 32 m of reperfusion. Relative ATP content in each heart during the pre-ischemic, ischemic, and reperfusion periods was determined from the signal intensity of the β-phosphate resonance signal at -16 ppm. Time-averaged spectra were collected at 243 MHz from the intact beating hearts in 4 m time blocks using 64 scans with a 45° flip angle and 2 s interpulse interval, using previously described data collection and processing/analysis schemes for high-energy phosphate levels and intracellular pH [15–17].

### Y2H screen and HspB2 dependency tests

Yeast harboring a human cardiac cDNA Y2H library (Clontech) were mated by the standard Matchmaker mating protocol (Clontech) to yeast harboring a full-length human *HSPB2* plasmid (pJG591). The bait plasmid (pJG591) was constructed by standard cloning techniques in which *HSPB2* was amplified by PCR from a human template using primers GGCGAATTCTCGGGCCGCTCAGTGCCAC and GGCGGATCCTCAGGGCTCAACTATGGCTGCC and cloned into the EcoR1/BamHI sites of pGBKT7 (Clontech) for fusion to the Gal4 DNA binding domain at the N-terminus; the C-terminus is native. Over 22 million yeasts were mated, from which 10,000 colonies arose on SD-Trp-Leu plus Aureobasidin (Clontech) selection plates. Colonies were then patched to alternate Y2H selection plates (SD-Trp-Leu-His-Ade) for phenotype validation through alternate transcriptional reporters (HIS and ADE). The plasmid inserts of the library were identified for over 1,000 colonies arising on the SD-Trp-Leu-His-Ade plates by colony PCR (primer agatggtgcacgatgcacag and ctattcgatgatgaagataccccacc) followed by sequencing and NCBI BLASTN [18] analysis. A subset of library plasmids were purified and secondarily subjected to false positive analysis. Briefly, plasmids were retransformed into yeast harboring the *HSPB2*-bait plasmid or empty bait plasmid, selected on SD-Trp-Leu and streaked to SD-Trp-Leu-His-Ade to test for an *HSPB2*-dependent interaction. For HspB2 specificity testing, *HSPB5* was PCR amplified from a human template and cloned into the EcoRI/SalI sites of pGBKT7 (Clontech) using primers GGCGAATTCGACATCGCCATC CACCACCCC and GGCGTCGACCTATTTCTTGGGGGCTGCGGTGAC. The *HSPB5* bait plasmid was then co-transformed with a library plasmid into yeast, selected on SD-Trp-Leu and streaked to SD-Trp-Leu-His-Ade to test for an *HSPB5*-dependent interaction.

### Mitochondrial co-IP proteomics

Mitochondrial HspB2 was immunopurified from mice harboring either the wild type *HSPB2* (HSPB2wt), transgenic cardiac *HSPB2* (HSPB2cTg), or an *HSPB2* cardiac knockout (HSPB2cKO, [9]). For each group, mitochondria from four mouse hearts were combined, lysed in 0.1% NP-40 homogenization buffer, and 2 mg was incubated with anti-HspB2 antibodies [9]. The IP eluates were then fractionated on SDS-PAGE, excised and analyzed by LC-MS/MS. Samples had one biological replicate and two technical replicates. Technical replicates showed greater than 90% overlap. The raw data were analyzed by BioWorks (ThermoFisher Scientific, version 3.3.1 SP1) and proteins were identified using SEQUEST (ThermoFisher Scientific, version 3.3.1) and Scaffold (Proteome Software, version 3.3.3). At least two peptides and 99.0% protein confidence were required for protein algorithms; the global false discovery rate was approximately 0.1%.

### Bioinformatic analysis of the HspB2 interactome

Cytoscape version 3.2.1 [19] was used to construct protein-protein interaction networks by retrieving previously identified interactions from the *mentha*, Reactome-Fls, Reactome, IntAct and MINT databases (partners reported for either human or mouse proteins) and mapping them into a single merged network. Gene Ontology analysis for the resulting networks was done using the Cytoscape ClueGO plugin [20]. Associated diseases were obtained using the Online Mendelian Inheritance in Man (OMIM) database (McKusick-Nathans Institute of Genetic Medicine, Johns Hopkins University (Baltimore, MD). World Wide Web URL: http://omim.org/).

### Protein purification and *in vitro* chaperone assays

*GAPDH* and *HSPB2* were cloned into pet15b (Novagen) for expression and purification. *GAPDH* was amplified from Origene plasmids SC118869 (accession number NM_002046) using the following primers, GGCCATATGGGGAAGGTGAAGGTCGGAGTC and GGCGGATCCTTACTCCTTGGAGGCCATGTGGGC, and cloned into pet15b at the NdeI/BamHI sites. *HSPB2* was PCR amplified from pJG591 (using primers GGCCATATGTCGGGCCGCTCAGTGCC and GGCGGATCCTCAGGGCTCAACTATGGCTGCC) and cloned into pet15b at the NdeI/BamHI sites. Expression vectors were transformed into BL21 (DE3) *E*. *coli* (Novagen). Overnight cultures of transformants were diluted 1:500 into LB-AMP and grown for 3 h at 37°C. Expression was induced by 0.5 mM ITPG for 5 h at 37°C. Pelleted bacteria were then resuspended in 20 mL XWA lysis buffer (20 mM HEPES pH 7.4, 10 mM KCl, 1.5 mM MgCl_2_, 1 mM EDTA pH 8.0, 1 mM EGTA, 300 mM NaCl, 1 mM beta-mercaptoethanol, pH 7.4, and Roche Complete Protease Inhibitor Cocktail). Cells were lysed using the Microfluidics M-110P homogenizer and debris pelleted at 15,000 rpm for 60 m. The supernatants were incubated with 250 μL of Ni-NTA agarose (Qiagen) for 2–3 h, beads were washed twice with 15–25 mL of XWA lysis buffer and then transferred to a column to be washed with 50 mL of lysis buffer containing 25 mM imidazole. Protein was eluted three times per sample with 0.3 mL of lysis buffer containing 250 mM imidazole and only 100 mM NaCl. The control, porcine citrate synthase was purchased from Sigma-Aldrich. Proteins were dialyzed into 40 mM HEPES-KOH buffer (pH 7.5) prior to *in vitro* chaperone assays. *In vitro* chaperone assays were conducted as previously described [21] by incubating 0.015 mM GAPDH with and without 0.105 mM HspB2 at high temperature (43°C) for 30 m in 40 mM HEPES-KOH buffer (pH 7.5). Seven-fold excess of chaperone compared to substrate is used because oligomerization is required for activity. Aggregates were then pelleted by centrifugation (13,000 rpm for 10 m in a microcentrifuge). For chaperone assays, both supernatant and pellet were analyzed by SDS-PAGE. Bands of the SDS-PAGE gel were analyzed using ImageJ software [22], and pellet to supernatant ratios calculated. Samples were run in triplicate and averaged.

### *In vivo* chaperone assays

*In vivo* GAPDH chaperone activity assays were performed by transfecting plasmids bearing human HspB2 fused to myc peptide or the empty plasmid control (pCMV-myc) into C2C12 cells in a 6-well plate using 6.5 μL lipofectamine 2000 (Life Technologies) and manufacturers protocol. After 48 h at 37°C, cells were shifted to 45°C for 30 m and then placed back at 37°C for three h prior to lysis. Cells were resuspended in 300 μL lysis buffer (20 mM HEPES (pH 7.8), 10 mM KCl, 100 mM NaCl, 1 mM EDTA, 1 mM EGTA, 1% NP-40 and 10% glycerol), homogenized in a Dounce homogenizer ten times for five seconds on ice, and debris pelleted at 13,000 rpm for 5 m. The pellet was resuspended in 100 μL of 2X SDS buffer (125 mM Tris-Cl, pH 6.8, 0.01% (w/v) bromophenol blue, 100 mM fresh dithiothreitol, 2.5% (w/v) SDS, 25% glycerol) while 6X SDS buffer was added to the supernatant for a final concentration of 1X. Samples were boiled 2 m, analyzed by 12% SDS-PAGE and transferred to nitrocellulose paper. Proteins were visualized using anti-GAPDH antibody, anti-beta-Tubulin antibody, and anti-Myc antibody (all antibodies are from Cell Signaling Technologies). Protein bands were analyzed using ImageJ software [22] and pellet to supernatant ratios were calculated.

### Data analysis, statistics and repository

Data are expressed as average ± standard deviation with student's *t-*test used for statistical analysis using two-tailed distribution and two-sample equal/unequal variance for all experiments with the exception of the NMR experiments, which were analyzed by ANOVA with Student-Newman Keuls post test [23]. Statistical significance was set at *p*<0.05. The HspB2 interactome described herein has been deposited in the Biological General Repository for Interaction Datasets (BioGrid, [24]) where it may be identified by PubMed ID.

### Ethics statement

This study was carried out in strict accordance with the Guide for the Care and Use of Laboratory Animals of the National Institutes of Health. The protocol was approved by the Institutional Animal Care and Use Committee (IACUC) at the University of Utah (Permit Number: 10–05006). All surgery was performed under ketamine and xylazine cocktail anesthesia administered intraperitoneally, and all efforts were made to minimize suffering.

## Results

### Cardiac HSPB2 OE does not alter levels of other sHSPs

Cardiac-specific *HSPB2* overexpressor mice (HSPB2cTg) were generated using human *HSPB2* under the control of α-myosin heavy chain (α-MyHC) promoter as schematically shown in Fig 1A. Neither early lethality nor age-related cardiomyopathy was observed in these HSPB2cTg mice (data not shown). In detergent soluble extracts, protein levels of HSPB2 were markedly elevated (~4 fold) in hearts of HSPB2cTg mice (n = 4 animals per group) compared with non-transgenic littermates (HSPB2NTg) (Fig 1B, quantification in Fig 1C). To determine the effects of HSPB2cTg OE, the expression patterns of several other sHSPs were analyzed by western blot. The previously reported sHSP binding partners of HspB2 are HspB3 [25, 26], HspB5 [27] and HspB8 [28]. Protein levels of HspB1, HspB3, HspB5, HspB6 and HspB8 were all found to be unaltered in the soluble fractions, whereas HspB6 was modestly *(P* = 0.035) and HspB8 was non-statistically significantly elevated *(P* = 0.064) in the detergent insoluble fraction, respectively (Fig 1B and 1C). In contrast, protein levels of HspB1, HspB3 and HspB5 were unaffected by cardiac-specific *HSPB2* OE in intact mouse hearts.

### HSPB2 OE protects mouse hearts from I/R injury

**Fig 2:**
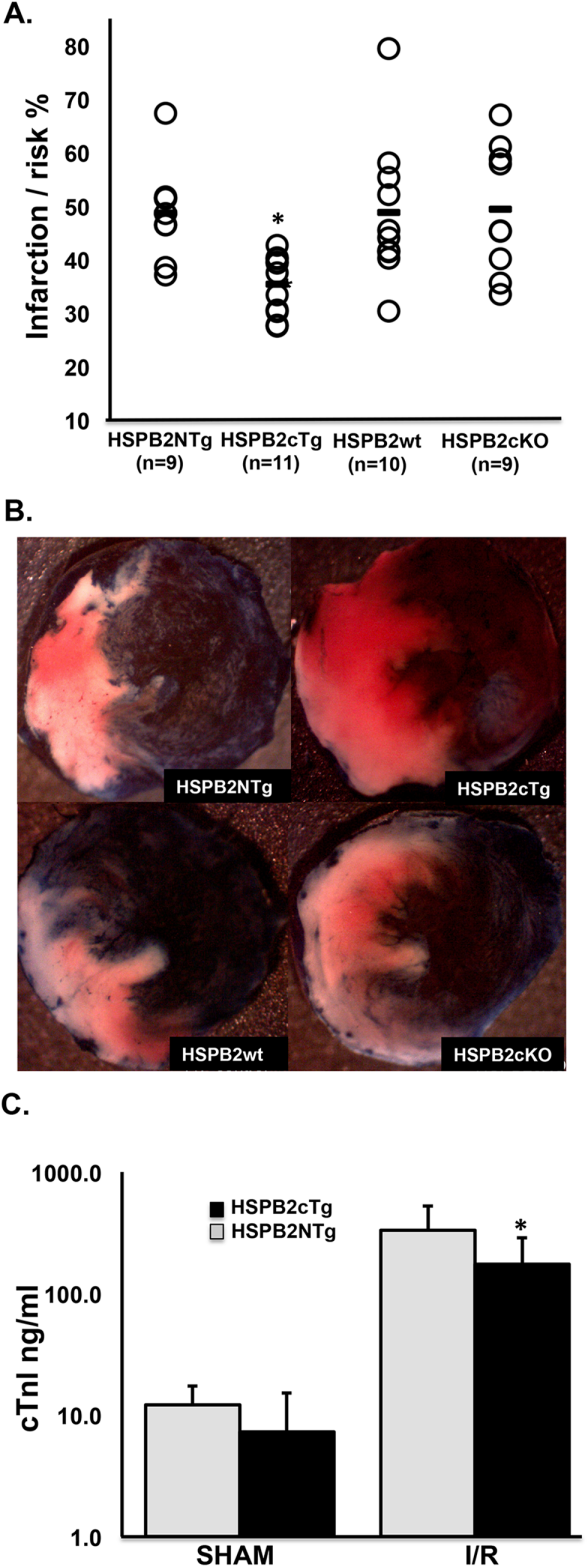
*HSPB2* OE protects mouse hearts from ischemia/reperfusion (I/R) injury *in vivo*. **(A)** Quantification of mouse cardiac infarction/risk size 24 h post I/R surgery. Each circle represents one animal from the transgenic cardiac overexpressor (HSPB2cTg), the cardiac knockout (HSPB2cKO), or control groups (HSPB2NTg and HSPB2wt, respectively). The number of animals in each group is labeled in the figure. **(B)** Representative staining images of mouse cardiac infarction post I/R surgery indicating the decreased infarct size in HSPB2cTg animals compared to controls. The same groups of animals as A are shown. Non-risk, live, and infarction tissue stained dark blue, red and white respectively. I/R surgery and infarction size measurements were performed as described in *Material and Methods*. The duration of ischemia lasted 45 m before deflation of the balloon and suture tie to initiate reperfusion. Hearts were excised 24 h after reperfusion start, cannulized, and the aorta was perfused with 5% Evans Blue, which stained the non-risk area dark blue. **(C)** Cardiac specific troponin-I levels in mouse serum 24 h post I/R surgery indicate HSPB2 OE is cardioprotective. Mouse serum was diluted in series from 1:10 to 1:1000, and loaded into a 96-well sample plate from the High Sensitive Mouse Cardiac Troponin-I kit (Life Diagnostics, Inc.). Troponin I level was measured at 450 nm by following the manufacturers recommendations. * *P*<0.05 when compared with corresponding HSPB2NTg group.

We have reported paradoxical effects of either *HSPB2* KO and/or *HSPB2*/*HSPB5* DKO in I/R studies [13, 29]. To investigate the specific role of HSPB2 in I/R injury, transgenic mice overexpressing cardiac *HSPB2* (HSPB2cTg) were compared with cardiac *HSPB2* KO mice (HSPB2cKO) and their littermate controls (HSPB2NTg and HSPB2wt respectively, n = 9–11 per group). Mouse hearts of HSPB2cTg (n = 11), HSPB2cKO (n = 9) and their corresponding controls underwent 45 m of ischemia followed by 24 h of reperfusion *in situ*. The size of infarction in each mouse heart was analyzed as described in Materials and Methods. An infarction area of 35.4 ± 5.6% was significantly lower in HSPB2cTg mouse hearts compared with the infarction area of 48.7 ± 10.0% for the HSPB2NTg controls (p< 0.0006, Fig 2A and 2B). Both HSPB2cKO and the corresponding Flox control animals (HSPB2wt) developed similar sizes of heart infarction upon I/R *in situ*, 49.4 ± 12.1% vs. 48.8 ± 13.5% respectively. Of all cardiac markers, cardiac specific troponin-I is the most sensitive indicator for cardiomyocyte injury, and its amount in the blood correlates well with heart infarction size after microvascular obstruction [30]. In agreement with the smaller cardiac infarction size, HSPB2cTg animals had significantly lower troponin-I levels after I/R stress compared with HSPB2NTg controls (Fig 2C). Troponin-I levels were similar between HSPB2cTg and HSPB2NTg after sham surgery in these animals.

In addition to infarct size, the cardiac function of these animals was assessed with both echocardiography and left ventricle catheter (Table 1). Interestingly, echocardiographic studies revealed wall motion abnormality at 24-h time points post I/R in HSPB2NTg but not in HSPB2cTg animals, in support of smaller infarction size in the HSPB2cTg animals. In addition, cardiac ejection fraction appeared to be reduced only in the HSPB2NTg group, although the data did not achieve statistical significance. The hemodynamic data acquired by left ventricle catheter did not show any difference among groups.

**Table 1:** Heart function in HSPB2cTg mouse with ischemia/reperfusion injury *in vivo*. Hemodynamic data is acquired by left ventricle catheter and analyzed by LabChart 7. Echocardiography is acquired with VisualSonics Vevo2100 imaging system. LV, left ventricle; SP, systolic pressure; DP, diastolic pressure; EDP, end diastolic pressure; HR, heart rate; LVEF, left ventricle ejection fraction; IR, ischemia/reperfusion. Each experiment group acquired by LV catheter contains at least 6 mice. Statistics and calculation of +dp/dt, -dp/dt and LVEF are described in *Materials and Methods*.

Groups	Sham		I/R	
	HSPB2NTg	HSPB2cTg	HSPB2NTg	HSBP2cTg
**LV catheter**				
Aorta SP	88.57 ± 23.28	76.41 ± 13.03	73.85 ± 6.38	72.35 ± 6.57
Aorta DP	61.90 ± 11.84	49.25 ± 20.21	50.43 ± 8.53	48.94 ± 9.36
LV SP	89.56 ± 19.61	78.74 ± 12.74	76.79 ± 6.12 *	72.89 ± 5.75
LV EDP	1.96 ± 2.17	2.15 ± 1.75	5.89 ± 2.61	4.97 ± 2.52
HR	328.37 ± 45.02	347.14 ± 39.60	335.02 ± 44.87	345.13 ± 59.35
+dp/dt	5834.53 ± 1650.23	6159.39 ± 2848.88	5196.99 ± 606.56	4785.15 ± 1140.38
-dp/dt	-4867.77 ± 1716.10	-5475.04 ± 1873.88	-4169.81 ± 565.11	-4220.02 ± 1011.12
**Echo**				
LVEF	0.700342461	0.78	0.66 ± 0.15	0.81 ± 0.12
Wall motion abnormality	0/1	0/1	3/8	0/4

### Mitochondria function in HSPB2cTg mouse heart with I/R injury

**Fig 3:**
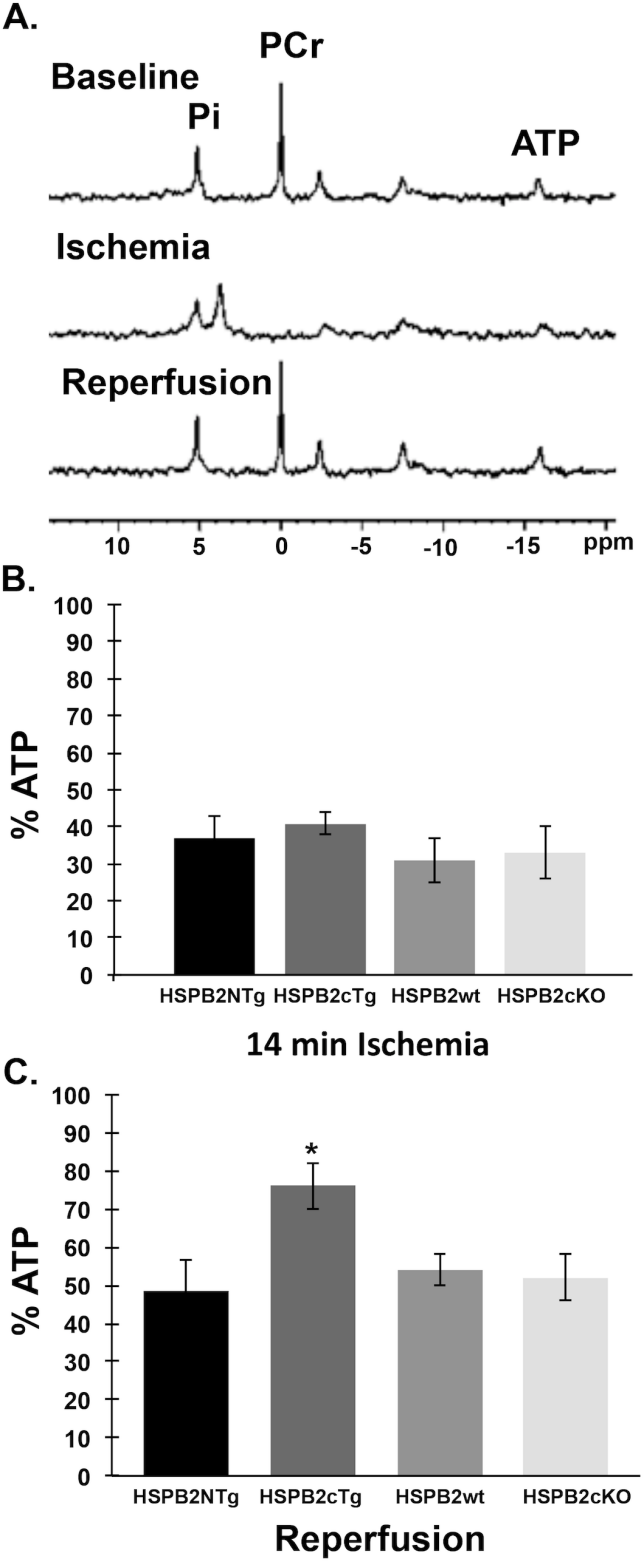
Mitochondrial protection in HSPB2cTg mouse heart with ischemia/reperfusion (I/R) injury. **(A)** Representative ^31^P NMR spectra of a transgenic HSPB2 overexpressor (HSPB2cTg) heart taken at baseline before ischemia, at 14 m of ischemia, and at 32 m of reperfusion. Phosphocreatine (PCr) was depleted during ischemia but rapidly recovered upon reperfusion in all groups. Note loss and recovery of ATP content, as represented by the only resonance that is uncontaminated from signal from ADP and AMP, which is the β-phosphate group at -16 ppm. The peak at 3.5 ppm is signal from the intracellular inorganic phosphate (Pi), which shifts to lower values due to the increasing concentration of diprotonated Pi versus mono-protonated Pi as the pH becomes increasingly acidic during ischemia. The peak at 5 ppm is a combined signal from both extracellular pH and residual buffer surrounding and in the isolated perfused heart, which also contains inorganic phosphate and reflects neutral pH. **(B)** ATP content after 14 m of ischemia was reduced similarly in all groups, as shown as a percentage of pre-ischemic content at 100%. (C) Recovery of ATP content upon reperfusion was greater in HSPB2cTg hearts than in the knockout hearts (HSPB2KO) or control groups (HSPB2NTg or HSPB2wt, respectively). Values shown are at 2 min reperfusion and did not change significantly from these over the remaining duration of the reperfusion period. * *P*<0.05 when compared with corresponding HSPB2NTg group.

We previously reported a deficit in the recovery of phosphocreatine (PCr) and ATP levels in the *HSPB2*-deficient mice following I/R stress, suggesting HspB2 protects mitochondrial function during I/R [9]. The ability of *HSPB2* OE to protect ATP synthesis was formally determined throughout the I/R protocol for each experimental group (HSPB2cTg, n = 7, HSPB2NTg, n = 5, HSPB2cKO, n = 7, and HSPB2wt, n = 6). Fig 3A–3C displays representative changes in NMR-detected high-energy phosphate compounds and comparisons of ATP content among groups at the end of ischemia and upon reperfusion. Although no difference in PCr content occurred among experimental heart groups at any time throughout the protocol, consistent with the role of PCr as a phosphagen that responds to reoxygenation of viable mitochondria following non-lethal ischemia, we did observe a difference in ATP content in the HSPB2cTg mice.

As shown in Fig 3B, there was no significant difference in ATP loss during the ischemic period, demonstrating that all groups held similar susceptibility to impaired energy production during zero flow ischemia. Since oxidative mitochondrial energy production was essentially shut down during ischemia, the presence or absence of HSPB2 did not affect ATP levels during ischemia. ATP depletion was required to support the same energy demands for residual cell function during the ischemic insult among all experimental groups. The similar levels of ATP hydrolysis (ATP + H_2_O → ADP + Pi + H^+^) that occurred during ischemia among all groups is consistent with the similar reduction in intracellular pH among the groups, from a preischemic baseline of 7.1 to the final pH range observed at the end of ischemia of 6.2–6.1. The peak at 3.5 ppm is signal from the intracellular inorganic phosphate (Pi), which shifts to lower values due to the increasing concentration of diprotonated Pi versus mono-protonated Pi as the pH becomes increasingly acidic during ischemia. The peak at 5 ppm is a combined signal from both extracellular pH and residual buffer surrounding and in the isolated perfused heart, which also contains inorganic phosphate and reflects neutral pH.

In contrast to the similarities observed during ischemia, ATP content and recovery was augmented in HSPB2cTg hearts upon early reperfusion in comparison to all other groups (Fig 3C, ANOVA with Student-Newman Keuls post test). These data show a 41% to 57% increase in the postischemic mean ATP content of the HSP2cTg hearts over the other groups. The initial ATP content seen upon reperfusion persisted throughout the entire assay period in all groups, with no significant change over time (Repeated Measures ANOVA). These results strongly support a role for *HSPB2* alone via cardioprotection during ischemia and sustained mitochondrial function upon reperfusion.

Kadono et al. suggests that the lack of *HSPB2* and *HSPB5* preconditions myocytes for damage due to increased calcium sequestration by the mitochondria, a known step in the inducement of mitochondria permeability transition [31, 32]. Mitochondrial swelling assays were performed in the HSPB2cTg and HSPB2NTg mice to determine if this calcium response is dependent on HspB2. Although mitochondria isolated from HSPB2cTg hearts (n = 4) trended toward lower levels of swelling in response to calcium, the differences were not significant when analyzed by student’s t-test (S1 Fig).

### An unbiased Y2H screen for HspB2 substrates reveals surprising specificity for mitochondrial binding partners

The foregoing findings for HspB2-associated phenotypes predicts protective roles for HspB2 activators and strongly suggest that HspB2 has hitherto unknown molecular clientele critical to mitochondrial health, however mitochondrial HspB2 clientele are unreported. To identify these client proteins, we performed an unbiased, large-scale Y2H screen using a human heart cDNA Y2H library (Clontech) as prey and human *HSPB2* as bait. From over 22 million mated yeasts, approximately 10,000 putative interactions were detected indicating only one of every ~2,000 mattings resulted in a positive Y2H interaction, suggesting a high degree of specificity of *HSPB2* targets. Over 1,000 library plasmids were re-plated for phenotypic verification and subsequently sequenced to identify the compendium of interacting partners. As expected for a molecular chaperone, such sequencing revealed a large number of interacting partners (437 unique hits). These binding partners were further grouped by their frequencies, depending on if they were retrieved only once (S1 Table, “single-hit” list) or multiple times (Table 2, “multi-hit” list) in the Y2H screen.

**Table 2:** Putative HspB2 binding partners retrieved from the Y2H screen reveal a role in muscle and mitochondrial maintenance. Proteins retrieved multiple times from a Y2H screen for HspB2 binding partners (84 hits) are given along with the following information: protein name, UniProt [73, 74] protein accession number (hyperlink), gene symbol, primary cellular localization reported on HPRD [76] or UniProt, the number of times it was retrieved from the screen (# hits) and the amino acids corresponding to the library construct from the n-terminal amino acid to the c-terminal amino acid (if the terminal amino acid is not reached the total amino acids are given in parenthesis). In some cases the nucleotide fusion occurred before the initiating ATG and is indicated by the number of amino acids upstream of the start (for example, -12–136 for LGALS1 denotes 22 amino acids upstream of the ATG start to amino acid 136). Hits that did not correspond to a coding sequence are not listed and include the 3’ region of ABLIM1, AXIN1, DYRK1A, FHL1, GPX3, HIPK2, NCAM1, PPAPDC3, PLXNA4, LRP10, SCN1B, SEC62, SGSM2, SLC25A4, SNTA1, SYNPO, TNIP1, USP28, and XIRP1 mRNA. The proteins are functionally categorized by their description on UniProt [73, 74] and/or GeneCards [73]. See supplementary S1 Table for a complete list of putative HspB2 binding partners retrieved from the Y2H screen only once.

Protein Name	UniProt Accession	Gene Symbol	Cellular Localization**	# hits	aa
**Myofibril or Cytoskeleton Structure/Regulation**					
Actin, alpha 1, skeletal muscle*	P68133	ACTA1	Cytoskeleton	8	253–377[Table-fn t002fn003]
Actin, beta	P60709	ACTB	Cytoskeleton	2	267–375[Table-fn t002fn003]
Actin, alpha, cardiac muscle 1	P68032	ACTC1	Cytoskeleton	37	84–377[Table-fn t002fn003]
Actin, gamma 1	P63261	ACTG1	Cytoskeleton	5	195–375[Table-fn t002fn003]
Capping protein (actin filament) muscle Z-line, alpha 2	P47755	CAPZA2	Cytoskeleton	3	117–286[Table-fn t002fn003]
Cardiomyopathy associated*	Q8N3K9	CMYA5	Cytoskeleton	4	3972–4069[Table-fn t002fn003]
Biglycan	P21810	BGN	Extracellular	2	191–368[Table-fn t002fn003]
Dynactin 1*	Q14203	DCTN1	Cytoskeleton	3	910–1144[Table-fn t002fn003]
Beta-enolase 3 (beta, muscle)*	P13929	ENO3	Cytoskeleton	7	85–434[Table-fn t002fn003]
Filamin C, gamma	Q14315	FLNC	Cytoskeleton	2	2608–2692[Table-fn t002fn003]
Myosin binding protein C, cardiac*	Q14896	MYBPC3	Cytoskeleton	11	945–1274[Table-fn t002fn003]
Myosin, heavy chain 6, cardiac muscle, alpha*	P13533	MYH6	Cytoskeleton	3	846–958[Table-fn t002fn004]
Myosin, heavy chain 7, cardiac muscle, beta*	A5YM51	MYH7	Cytoskeleton	14	1865–1935[Table-fn t002fn003]
Myomesin (M-protein) 2*	P54296	MYOM2	Cytoskeleton	6	1283–1465[Table-fn t002fn003]
Ryanodine receptor 2 (cardiac)	Q92736	RYR2	SR	3	1864–2359(4968)^#^
Titin-cap (telethonin)	O15273	TCAP	Cytoskeleton	6	92–167[Table-fn t002fn003]
Titin*	Q8WZ42	TTN	Cytoskeleton	5	1493–1890(5605)
Troponin I type 3*	Q6FGX2	TNNI3	Cytoskeleton	5	-17–210[Table-fn t002fn003]
WD repeat domain 1	O75083	WDR1	Cytoskeleton	3	221–466^#^
**Fermentation/Respiration**					
Acetyl-CoA acyltransferase 2*	P42765	ACAA2	Mito IM	5	208–397[Table-fn t002fn003]
Aldehyde dehydrogenase, mitochondrial	P05091	ALDH2	Mito Matrix		266–470[Table-fn t002fn003] ^#^
Aldolase A, fructose-bisphosphate*	P04075	ALDOA	Cytoplasm	15	159–418[Table-fn t002fn003]
ATP synthase subunit alpha, mitochondrial, F1*	P25705	ATP5A1	Mito IM/Matrix	11	137–503[Table-fn t002fn003]
Cytochrome P450, family 1, subfamily B, polypeptide1	Q16678	CYP1B1	ER	4	436–543[Table-fn t002fn003]
Enolase 1, alpha-enolase	P06733	ENO1	Cytoskeleton	4	-36–341^#^
Electron-transfer-flavoprotein, alpha subunit*	P13804	ETFA	Mito Matrix	15	108–284[Table-fn t002fn003]
Glyceraldehyde 3-phosphate dehydro.*	P04406	GAPDH	Cytoplasm	19	21–293[Table-fn t002fn003]
Hydroxyacyl-CoA dehydro./3-ketoacyl-CoA thiolase*	P55084	HADHB	Mito IM/OM	5	205–452[Table-fn t002fn003] ^#^
Methylcrotonoyl-CoA carboxylase beta chain	Q9HCC0	MCCC2	Mito Matrix	2	42–563[Table-fn t002fn003]
Malate dehydro. 1, NAD (soluble)	P40925	MDH1	Mito, Cytoplasm	2	68–352[Table-fn t002fn003] ^#^
Cytochrome c oxidase subunit 1	P00395	MT-CO1	Mito IM	12	222–512[Table-fn t002fn003]
Cytochrome c oxidase subunit 2	P00403	MT-CO2	Mito IM	2	67–227[Table-fn t002fn003]
Cytochrome c oxidase subunit 3	P00414	MT-CO3	Mito IM	2	1–260[Table-fn t002fn003]
NADH dehydro. (ubiquinone) 1 alpha subcomplex 6*	P56556	NDUFA6	Mito IM	2	66–154[Table-fn t002fn003]
NADH dehydro. (ubiquinone) 1 alpha subcomplex, 13	Q9P0J0	NDUFA13	Mito IM	2	-5–144[Table-fn t002fn003]
Oxoglutarate (alpha-ketoglutarate) dehydro.*	A2VCT2	OGDH	Mito	2	907–1019[Table-fn t002fn003]
Phosphoglucomutase 1	P36871	PGM1	Cytoplasm	2	250–365[Table-fn t002fn003] ^#^
Succinate dehydro. complex, subunit A*	D6RFM5	SDHA	Mito IM	5	351–654[Table-fn t002fn003]
**Signal Transduction**					
Ankyrin repeat & GTPase Arf activating protein 11	Q8TF27	AGAP11	Cytoplasm	2	19–550[Table-fn t002fn003] ^#^
DNA-damage-inducible transcript 4	Q9NX09	DDIT4	Cytoplasm	2	167–232[Table-fn t002fn003]
Dual specific phosphatase 1	P28562	DUSP1	Nucleus	2	158–367[Table-fn t002fn003]
Four and a half LIM domains 2*	Q14192	FHL2	Nucleus	10	47–279[Table-fn t002fn003]
Myozenin 2	Q9NPC6	MYOZ2	Cytoplasm	2	31–264[Table-fn t002fn003] ^#^
Parvin, alpha	Q9NVD7	PARVA	PM	2	181–412[Table-fn t002fn003]
Prosaposin*	Q5BJH1	PSAP	Lysosome	14	425–526[Table-fn t002fn003]
Zinc finger protein 335*	Q8IW09	ZNF335	Nucleus	2	610–1342[Table-fn t002fn003] ^#^
**Gene/Protein Expression**					
Brain expressed X-linked 4	Q9NWD9	BEX4	Cytoplasm	2	1–120[Table-fn t002fn003]
Eukaryotic translation elongation factor 1 alpha 1*	P68104	EEF1A1	Ribosome, Nucleus	13	213–462[Table-fn t002fn003] ^#^
Eukaryotic translation elongation factor 2	Q6PK56	EEF2	Ribosome	2	685–858[Table-fn t002fn003]
Eukaryotic translation initiation factor 4A2	Q14240	EIF4A2	Ribosome	3	360–407[Table-fn t002fn003]
Glycine-tRNA synthetase	P41250	GARS	Cytoplasm	2	254–739[Table-fn t002fn003] ^#^
Asparagine-tRNA ligase	O43776	NARS	Cytoplasm	2	379–548[Table-fn t002fn003]
NHP2 non-histone chromosome protein 2-like 1	P55769	NHP2L1	Nucleus	2	41–123[Table-fn t002fn003] ^#^
Ribosomal protein L11, 60S*	P62913	RPL11	Ribosome	10	83–177[Table-fn t002fn003]
Ribosomal protein L36a, 60S*	J3KQN4	RPL36A	Ribosome	13	81–142[Table-fn t002fn003]
Ribosomal protein L36a-like, 60S	Q969Q0	RPL36AL	Ribosome	2	45–106[Table-fn t002fn003]
Ribosomal protein S11, 40S	P62280	RPS11	Ribosome	4	90–158[Table-fn t002fn003]
THUMP domain-containing 2	Q9BTF0	THUMPD2	Cytoplasm	2	11–503[Table-fn t002fn003] ^#^
**Transport**					
ATP-binding cassette, sub-family C, member 9	O60706	ABCC9	PM	2	1263–1549[Table-fn t002fn003] ^#^
ATP-binding cassette, sub-family D, member 4	O14678	ABCD4	Peroxisome	2	204–606[Table-fn t002fn003]
ATPase, H+ transporting, V1 subunit E1	Q53Y06	ATP6V1E1	Lysosome	2	165–196[Table-fn t002fn003] ^#^
Myoglobin	P02144	MB	Cytoplasm	3	9–154[Table-fn t002fn003]
Protein phosphatase, IK	Q8N3J5	PPM1K	Mito Matrix	2	260–372[Table-fn t002fn003]
Stromal cell-derived factor 2	Q99470	SDF2	Extracellular	2	(-9)–211
Solute carrier family 2, member 1	P11166	SLC2A1	PM	3	369–492[Table-fn t002fn003]
**Protein Degradation**					
Alpha-2-macroglobulin	P01023	A2M	Extracellular	3	1184–1474[Table-fn t002fn003] ^#^
Cathepsin B	P07858	CTSB	Lysosome	4	238–339[Table-fn t002fn003]
Cathepsin D*	P07339	CTSD	Lysosome	4	229–413[Table-fn t002fn003]
**Nucleotide Metabolism**					
Creatine kinase, muscle	P06732	CKM	Cytoplasm	3	133–381[Table-fn t002fn003] ^#^
Guanylate kinase 1*	Q96IN2	GUK1	Cytoplasm	3	11–197[Table-fn t002fn003]
Putative deoxyribonuclease 1	Q6P1N9	TATDN1	Nucleus	2	-6–298[Table-fn t002fn003] ^#^
**Immune**					
Beta-2-microglobulin*	P61769	B2M	Extracellular	8	-11–119[Table-fn t002fn003]
Complement component 1, q subcomponent, A chain	P02745	C1QA	Extracellular	2	134–245[Table-fn t002fn003]
Thymocyte selection associated family member 2	Q5TEJ8	THEMIS2	Cytoplasm	2	246–447[Table-fn t002fn003]
**Cell Adhesion**					
Lectin, galactoside-binding, soluble, 1 (Galectin-1)*	P09382	LGALS1	Extracellular	9	-22–135[Table-fn t002fn003]
Plakophilin 2	Q99959	PKP2	Cytoskeleton’PM	3	619–837[Table-fn t002fn003]
**Osmoregulation**					
ATPase, Na+/K+ transporting, beta 1	Q6LEU2	ATP1B1	PM	2	101–303[Table-fn t002fn003]
**Small Molecule Biosynthesis**					
N-acylsphingosine amidohydrolase 1	Q13510	ASAH1	Lysosome	2	290–389[Table-fn t002fn003]
**Oxidoreductive Stress**					
Thioredoxin-interacting protein	Q9H3M7	TXNIP	Cytoplasm	6	175–391[Table-fn t002fn003]
**Chaperone**					
Heat Shock Protein 2 (HspB2)	Q16082	HSPB2	Cytoplasm	6	-66–175[Table-fn t002fn003]
Crystallin, alpha B*	P02511	HSPB5	Cytoskeleton	34	-3–175[Table-fn t002fn003]
**Unknown**					
Dynactin 6	O00399	DCTN6	Mito	3	82–190[Table-fn t002fn003]
Myeloid leukemia factor 2	Q15773	MLF2	Nucleus	2	98–248[Table-fn t002fn003] ^#^
Nucleotidase domain containing 2	Q9H857	NT5DC2	unknown	2	302–462[Table-fn t002fn003]

* Indicates the 30 proteins shown to be HspB2 dependent in the Y2H binding assay. All others were not tested for dependency. Of the total 49 plasmids tested for dependency from the multi-hitter list, 10 did not appear in frame from sequence analysis, 81.6% showed a positive Y2H result that was dependent on HspB2 (indicating a HspB2/prey interaction), 10.2% were false positives (they grew with either HspB2 or the empty bait plasmid), and 8.2% were true negatives (they did not grow with either HspB2 or the empty bait). Out of frame fusions, false positives and true negatives revealed through this analysis are not listed.

** Abbreviations include Endoplasmic Reticulum (ER), Mitochondrial (Mito), Mitochondrial Inner Membrane (Mito IM), Mitochondrial Outer Membrane (Mito OM), Mitochondrial Matrix (Mito Matrix), Plasma Membrane (PM) and Sarcoplasmic Reticulum (SR).]

Indicates that the final amino acid listed is the C-terminal amino acid of the native protein.

Indicates that there is extra DNA from the mitochondrial chromosome on the 3’ end of this construct in addition to the MYH6 DNA which adds an extra 82 aa.

# Indicates that the sequence quality was too poor to unambiguously tell the N-terminal fusion (WDR1, NHP2L, ATP6V1E1, TATDN1, MLF2) or the C-terminal end (RYR2, WDR1, ALDH2, ENO1, HADHB, MDH1, PGM1, AGAP11, MYOZ2, ANF335, GARS, CKM, ABCC9, EEF1A1, THUMPD2, A2M).

The advantage of high sensitivity in the Y2H method comes at the expense of specificity (i.e., high false positive rates) [12, 33, 34]. To test the overall reliability of the Y2H, a subset of the putative binding partners were tested for *HSPB2*-dependence. Briefly, prey plasmids encoding 90 of the putative HspB2 binding partners (49 from the multi-hit list and 41 from the single-hit list) were purified from yeast and reintroduced into naïve yeast harboring either the empty bait plasmid or the *HSPB2* bait plasmid. A remarkable 81.6% of the 49 multi-hit library plasmids tested showed a positive Y2H result that was dependent on *HSPB2* (i.e., growth with only the *HSPB2* bait, not the empty bait plasmid), 10.2% were false positives (i.e., growth either with or without HspB2), and 8.2% were true negatives (i.e., no growth with either HspB2 or the empty bait plasmid). In contrast, we found that only 51.2% of the 41 single hit plasmids harbored HspB2 dependent-binding partners, 12.2% were false positives, and another 36.6% were true negatives. The multi-hit binding partners with an 82% rate for validation were then sequenced to determine frame, and are used for all subsequent studies and analysis due to its reliability.

### The Y2H screen identifies non-redundant client proteins of cytosolic HspB2 and HspB5

**Fig 4:**
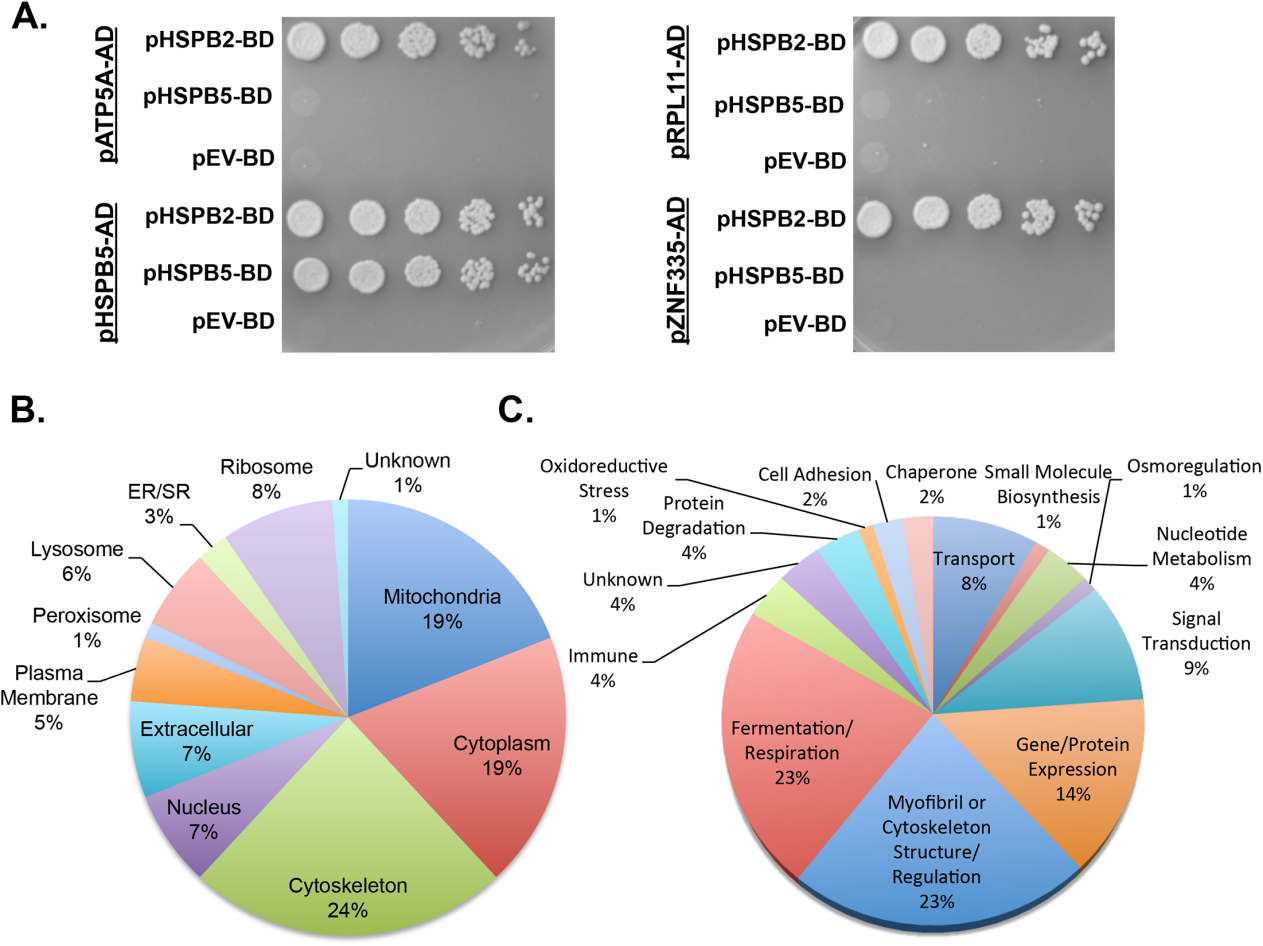
A large-scale HspB2 yeast two-hybrid (Y2H) screen reveals specificity in sHSP binding partners and identifies putative myofibril and mitochondrial clientele. **(A)** Y2H assays for direct binding between HspB2 or HspB5 baits and four putative HspB2 binding partner preys reveals non-redundant binding. Y2H prey plasmids (pGADT7, Clontech) retrieved from the HspB2 Y2H screen were transformed into Y2HGold cells (Clontech) containing bait plasmids (pGBKT7, Clontech) with either HSPB2 (pHSPB2-BD) or HSPB5 (pHSPB5-BD) fused to the Gal4 DNA binding domain (BD), or the empty vector (pEV-BD). Overnight cultures were grown for 48 h in SD-Trp-Leu to select for plasmids, then serially diluted 1:5 and plated on SD-Trp-Leu-His-Ade plates to select for protein-protein interaction. These four (ATP synthase alpha subunit (ATP5A1), alphaB-crystallin (HspB5), ribosomal protein L11 (RPL11), and zinc finger protein 335 (ZNF335) are representative. Results of all 12 binding partners are given in Table 3. **(B)** Pie charts summarizing the reported localization of the putative HspB2 binding partners revealed that a majority are localized to the cytoplasm, cytoskeleton or mitochondria. **(C)** Pie charts summarizing the reported function for putative cardiac HspB2 binding partners reveals two major categories of myofibril or mitochondrial function. Function and localization was assigned by basic description on GeneCards [73], UniProt [73, 74] or literature searches when necessary.

In order to test the specificity of the HspB2 Y2H interactome, the interactome was compared to the recently published HspB1 (Hsp27) interactome in which 228 binding partners were identified by Katsogiannou et al. from large-scale Y2H screens of testis and Hela cancer cell libraries [35]. Of the 84 proteins on the high confidence multi-hit HspB2 Y2H list, only two were identified as HspB1 binding partners (EIF4A2 and GARS), suggesting specificity of the HspB2 and HspB1 interactomes. However, different Y2H libraries were used in these studies that could also account for the variances. To further verify specificity of HspB2, a subset of HspB2 binding partners were tested for their ability to interact with a more closely related HSP, HspB5 (Fig 4A, Table 3). Remarkably, HspB2 was specific for a majority of the binding partners tested (eight of the 12 binding partners, or 67%), with only four displaying both HspB2 and HspB5 binding (including HspB5 itself, which was used as a positive control). In addition, two of these four HspB2 and HspB5 binding partners gave a much stronger Y2H result with HspB2 than HspB5. These findings validate the specificity of HspB2 in the large-scale Y2H screen and provide further *in vivo* support for non-redundant clientele among sHSPs, consistent with expression and phenotypic analyses that have revealed unique functions for HspB5 and HspB2 chaperones [6].

**Table 3:** HspB2 is highly specific for its targets as revealed by a comparison of HspB2 and HspB5 binding partners. Twelve binding partners from the HspB2 Y2H screen were tested for their ability to bind HspB2 or HspB5. The columns are as follows: gene (the name of the gene encoded on the library ‘prey’ plasmid retrieved from the Y2H screen), plasmid (the pJG number of the purified plasmid), HspB2 (the Y2H protein-protein interaction result using HspB2 as bait), HspB5 (the Y2H protein-protein interaction result using HspB5 as bait), and EV (the Y2H result using an empty vector control). Strong, medium or weak indicates apparent strength of the interaction as determined by growth on selective media (SD–Trp–Leu–His–Ade); NG means no detectable growth. HspB5 was used as a positive control and was also retrieved from the yeast two-hybrid screen.

**Gene**	**Plasmid**	**HspB2**	**HspB5**	**EV**
**HSPB5**	pJG933	Strong	Strong	NG
**ALDOA**	pJG774	Strong	Weak	NG
**ATP5A1**	pJG745	Strong	NG	NG
**DCTN1**	pJG885	Strong	Weak	NG
**ENO3**	pJG877	Strong	NG	NG
**EEF1A1**	pJG882	Strong	NG	NG
**MYH6**	pJG785	Strong	NG	NG
**OGDH**	pJG988	Strong	NG	NG
**PSAP**	pJG878	Strong	Strong	NG
**RPL11**	pJG903	Strong	NG	NG
**SDHA**	pJG871	Strong	NG	NG
**ZNF335**	pJG944	Strong	NG	NG

### The Y2H screen identifies mitochondrial clients for HspB2

The 84 high confidence binding partners identified by Y2H were further analyzed for their cellular localization and molecular functions. The majority of the binding partners have been reported to primarily localize to the mitochondria (19%), the cytoskeleton (24%) and the cytoplasm (19%) (Fig 4B). The functional classes to which the most interacting partners belong are those of fermentation/respiration (23%, including 13 targets directly involved in oxidative phosphorylation) and myofibril or cytoskeletal function (23%), consistent with the previously reported roles of HspB2 in ATP production [6, 9, 13] and muscle fiber maintenance [4, 6, 26, 29, 36, 37]. The high proportion of mitochondrial proteins identified by Y2H may, in part, be due to the abundance of mitochondria in cardiac tissue, yet there is interest in this subset of mitochondrial proteins when considering the total mitochondrial proteome, with over 1,000 mitochondrial proteins estimated in studies of both human [38] and rat [14] cardiac tissues.

### Coaffinity proteomics of HspB2 validates mitochondrial targets of HspB2

The identification of multiple mitochondrial binding partners poses a novel hypothesis that HspB2, a resident cytosolic molecular chaperone, might also have distinct mitochondrial clientele. Such clientele would be consistent with the transient association with the outer mitochondrial membrane reported by Nakagawa et al. from tissue culture studies [8]. To test this hypothesis, co-purification studies were performed followed by quantitative mass spectroscopy to identify both HspB2 mitochondrial localization as well as native mitochondrial binding partners [39, 40]. Anti-HspB2 antibodies [9] were used to IP protein from cardiac muscle mitochondrial lysate derived from either the HSPB2NTg control, heart-specific transgenic (HSPB2cTg), or heart-specific KO (HSPB2cKO) mice. Co-purified proteins were run on SDS-PAGE for fractionation and then identified by quantitative LC/MS/MS in two technical replicates. HspB2 was identified in the HSPB2cTg samples confirming mitochondrial association, but it was not identified either in the samples from littermate control (HSPB2NTg) or HSPB2cKO mice, presumably due to low or no HSPB2 expression, respectively. This restricted detection of HspB2 in only the OE lysates is consistent with previous assays for mitochondrial association of HspB2 and is thought to be due to low HspB2 expression [9].

**Fig 5:**
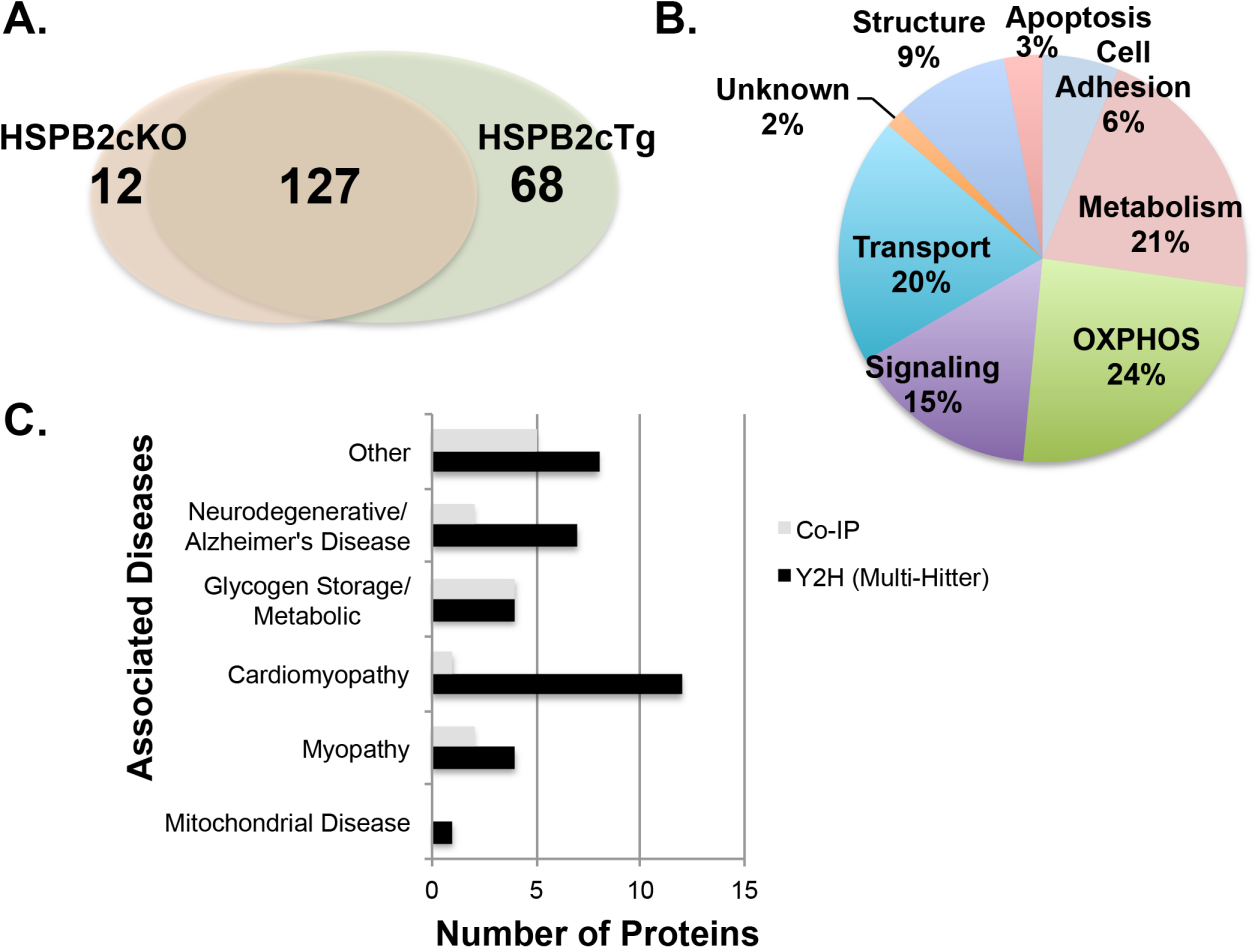
Co-purification of mitochondrial HspB2 binding partners supports the mitochondrial role predicted by Y2H and mouse data and identifies associations with disease. **(A)** Analysis of 207 proteins retrieved via co-immunoprecipitation (IP) from mouse cardiac mitochondria lysate with anti-HSPB2 antibodies reveals 68 proteins unique to the HSPB2cTg samples, 12 unique to the HSPB2cKO control and 127 that overlap. Mitochondrial HspB2 was immunopurified from mice harboring either transgenic cardiac HSPB2 (HSPB2cTg) or an HSPB2 cardiac knockout (HSPB2cKO). In each group, mitochondria were combined from four mouse hearts, lysed in 0.1% NP-40 homogenization buffer and 2 mg was incubated with anti-HspB2 antibodies. The IP eluates were then fractionated on SDS-PAGE, excised and analyzed by LC-MS/MS. Samples had one biological replicate and two technical replicates. **(B)** The unique proteins retrieved only from HSPB2cTg tissue (68 total) are enriched in oxidative phosphorylation functions. Predicted protein function was inferred by Gene Ontology [75]. **(C)** Diseases associated with proteins in the combined HspB2 Y2H and IP interactomes predict a role for HspB2 in neurodegenerative diseases and myopathies. Of the 149 HspB2 binding partners, 49 are associated with diseases and 57% of these are associated with myopathies and neurodegenerative diseases including cardiomyopathy and Alzheimer’s disease. Associated diseases were identified by the Online Mendelian Inheritance in Man (OMIM) database (McKusick-Nathans Institute of Genetic Medicine, Johns Hopkins University (Baltimore, MD). World Wide Web URL: http://omim.org/).

A total of 68 proteins (Table 4) were uniquely identified in the HSPB2cTg samples (Fig 5A). An additional 127 proteins were identified in both the HSPB2cTg and HSPB2cKO samples, suggesting they bind nonspecifically to the resins used in purification. The 68 HSPB2cTg-specific proteins compared with 12 HSPB2cKO-specific suggest HspB2-specific binding. In addition, the 68 HSPB2cTg-specific proteins were enriched for membrane bound proteins involved in oxidative phosphorylation, transport and metabolism (65%, Fig 5B), consistent with the Y2H results.

**Table 4:** Unique proteins identified from HSPB2 co-immunoprecipitation from HSPB2cTg heart tissue when compared to the HSPB2cKO heart tissue. This table lists information on all 68 uniquely identified proteins from the HSPB2cTg samples when compared with those identified from the HSPB2cKO samples by quantitative mass spectroscopy. It contains the following information sequentially: protein name, UniProt [73, 74] protein accession number, gene name, primary cellular localization from HPRD [76] or UniProt, molecular weight (MW), and protein length (# of amino acids). The proteins are functionally categorized by their description on UniProt and/or GeneCards.

Protein Name	UniProt Accession	Gene Symbol	Cellular Localization**	MW	Protein Length [67]
**Fermentation/Respiration**					
Protein Acad12	D3Z2B3	ACAD12	Mito	41332	373
ATP synthase subunit e, mitochondrial	P56385	ATP5I	Mito IM	8218	71
ATP synthase subunit g, mitochondrial	O75964	ATP5L	Mito IM	11407	103
CDGSH iron-sulfur domain-containing protein 1	Q9NZ45	CISD1	Mito OM	12079	108
Carnitine O-palmitoyltransferase 1, muscle isoform	A5PLL0	CPT1B	Mito,OM	88202	772
Carnitine palmitoyltransferase 2	P23786	CPT2	Mito IM	24889	223
Carnitine O-acetyltransferase	P43155	CRAT	Mito IM, ER, Perox	70823	626
Cytochrome c oxidase subunit 7A-related protein	Q6FGA0	COX7A2L	Mito IM	14913	134
Dihydrolipoyl dehydrogenase, mitochondrial	P09622	DLD	Mito Matrix	54254	509
Dihydrolipoyllysine-residue succinyltransferase	Q6IBS5	DLST	Mito, Nucleus	22212	454
Methylcrotonoyl-Coenzyme A carboxylase 2 (Beta)	Q9HCC0	MCCC2	Mito	61362	563
Novel protein GN = Mtfp1	Q9UDX5	MTFP1	Mito	18314	166
NADH-ubiquinone oxidoreductase chain 1	P03886	MT-ND1	Mito IM	36012	318
NADH-ubiquinone oxidoreductase chain 4	P03905	MT-ND4	Mito IM	51869	459
NADH-ubiquinone oxidoreductase chain 5	P03915	MT-ND5	Mito IM	68441	607
NADH dehydrogenase 1 alpha subcomplex subunit 11	Q86Y39	NDUFA11	Mito IM	14965	141
NADH dehydrogenase 1 beta subcomplex subunit 3	O43676	NDUFB3	Mito IM	11674	104
NADH dehydrogenase 1 beta subcomplex, 5	O43674	NDUFB5	Mito IM	14038	119
NADH dehydrogenase 1 beta subcomplex, 6	O95139	NDUFB6	Mito IM	15498	128
NADH dehydrogenase 1 beta subcomplex subunit 8	O95169	NDUFB8	Mito IM	21858	186
NADH dehydrogenase 1 beta subcomplex subunit 9	Q9Y6M9	NDUFB9	Mito IM	21966	179
NADH dehydrogenase 1 beta subcomplex, 11	Q9NX14	NDUFB11	Mito	17426	151
NADH dehydrogenase 1 subunit C2	O95298	NDUFC2	Mito IM	14146	120
NAD(P) transhydrogenase, mitochondrial	Q13423	NNT	Mito IM	113823	1086
2-oxoglutarate dehydrogenase, mitochondrial	Q02218	OGDH	Mito	117741	214
Ubiquinol-cytochrome c reductase hinge protein	P07919	UQCRH	Mito IM	10417	89
Cytochrome b-c1 complex subunit 8	O14949	UQCRQ	Mito IM	9751	82
**Transport **					
Platelet glycoprotein 4	P16671	CD36	Golgi, PM	52681	472
Mitochondrial carrier homolog 2	Q9Y6C9	MTCH2	Mito IM	32328	294
Metaxin 2	O75431	MTX2	Mito OM	29740	263
ADP/ATP translocase 2	Q6NVC0	SLC25A5	Mito IM	32915	298
Calcium-binding mitochondrial carrier protein Aralar1	O75746	SLC25A12	Mito IM	74554	677
Mitochondrial carnitine/acylcarnitine carrier protein	O43772	SLC25A20	Mito IM	33010	301
Solute carrier family 25 member 42	Q86VD7	SLC25A42	Mito IM	35241	318
**Cell Adhesion **					
Perlecan (Heparan sulfate proteoglycan 2)	Q2VPA1	HSPG2	Extracellular	469023	4735
Laminin subunit alpha-2	P24043	LAMA2	Extracellular	342761	3105
Laminin subunit gamma-1	P11047	LAMC1	Extracellular	177298	1607
Nidogen-1	P14543	NID1	Extracellular	136519	1245
Vinculin	P18206	VCL	Cytoplasm	116717	1066
**Small Molecule Biosynthesis and Catabolism **					
Apolipoprotein O	Q9BUR5	APOO	Mito	22587	198
Ferrochelatase (EC 4.99.1.1)	P22830	FECH	Mito, IM	44667	399
Amine oxidase [flavin-containing] B	P27338	MAOB	Mito OM	58541	520
Methylcrotonoyl-CoA carboxylase subunit alpha	Q96RQ3	MCCC1	Mito IM/Matrix	79327	717
Isoform 2 of Perilipin-4	Q96Q06	PLIN4	Cytoplasm,PM	139415	1403
**Gene/Protein Expression**					
Crip2 protein	P52943	CRIP2	Nucleus,Cortex	22709	208
Methylglutaconyl-CoA hydratase, mitochondrial	Q13825	AUH	Mito	22898	219
39S ribosomal protein L12, mitochondrial	P52815	MRPL12	Mito	21691	201
Nascent polypeptide-associated complex subunit alpha	Q13765	NACA	Cytoplasm,Nucleus	220499	2187
Sorting and assembly machinery component 50 homolog	Q9Y512	SAMM50	Mito, OM	51847	469
**Myofibril or Cytoskeleton Structure/Regulation **					
Alpha actinin 1a	P12814	ACTN1	Cytoplasm, Z-disk	102706	887
Spectrin alpha 2	A3KGU5	SPNA2	Cytoskeleton	282895	2457
Isoform 2 of Spectrin beta chain, brain 1	Q62261	SPNB2	Cytoskeleton	274223	2363
Troponin C, slow skeletal and cardiac muscles	P63316	TNNC1	Cytoskeleton	18421	161
Uncharacterized protein GN = Vim	P08670	VIM	PM	52185	453
**Intracellular Trafficking **					
AP-3 complex subunit delta-1	O14617	AP3D1	Cytoplasm,PM	135081	1199
EH-domain containing 4	Q9H223	EHD4	ER,PM	61481	541
Ras-related protein Rab-7a	P51149	RAB7A	Lysosome	23472	207
**Chaperone**					
Heat shock cognate 71 kDa protein	Q96IS6	HSPA8	Cytoplasm	70871	646
Heat shock protein beta-2	Q16082	HSPB2	Cytoplasm	20357	182
**Cell Death**					
Dynamin-like 120 kDa protein, mitochondrial	O60313	OPA1	Mito IM	78918	692
**Immune **					
Complement component 1 Q subcomponent-binding protein	Q07021	C1QBP	Mito Matrix	31013	278
**Oxidoreductive Stress**					
Apoptosis-inducing factor, mitochondrion-associated 1	O95831	AIFM1	Mito IM/OM	66748	612
**Unknown**					
Apolipoprotein O-like	Q6UXV4	APOOL	Mito IM	29244	265
Mitochondrial pyruvate carrier	Q9D023	MPC2	Mito IM	14269	127
ES1 protein homolog, mitochondrial	Q9D172	D10JHU81E	Mito	28072	266
Uncharacterized protein GN = Ogdhl	Q9ULD0	OGDHL	Cytoplasm	116584	1029
Prohibitin	P35232	PHB	Mito IM	29803	272
Translocase of inner mitochondrial membrane domain containing 1	Q9NPL8	TIMMDC1	Mito IM	31774	285

**Abbreviations include Endoplasmic Reticulum (ER), Mitochondrial (Mito), Mitochondrial Inner Membrane (Mito IM), Mitochondrial Outer Membrane (Mito OM), Mitochondrial Matrix (Mito Matrix), Peroxisome (Perox), Plasma Membrane (PM)

When combined, the 84 high confidence targets from the Y2H screen and 68 from co-IP studies identify 149 unique proteins as putative HspB2 clientele. Three proteins were identified by both methods (HspB2, OGDH, and MCCC2). The low level of overlap between the two independent screens is due to the difference in using whole-cell verses mitochondrial extract and is also consistent with previous reports on differences due to the diverse strengths of each approach [41]. Of note, if all of the proteins identified in the HSPB2cTg co-IP samples are analyzed (including the 127 that also occur in the HSPB2cKO control), there are 39 proteins that overlap between the Y2H and co-IP samples. These additional proteins interacted with the purification resin itself and may represent proteins that naturally aggregate easily, prime targets for chaperones. This aggregation, however, makes it difficult to show protein-protein association by co-IP and these proteins are not included in the 68 retrieved from co-IP.

### Network analysis of the combined HspB2 cardiac interactome reveals functional links to myopathies and neurodegenerative disease

HspB2 is one of only a few sHSP to date (including HspB9 and HspB10) without identified disease-associated human alleles. Therefore, the combined high confidence Y2H and co-IP interactome (a total of 149 unique proteins) was analyzed for diseases associated with the putative HspB2 binding partners. There were 35 Y2H and 14 co-IP HspB2 binding partners associated with disease, and of these, 28 (57%) were associated with myopathies and neurodegenerative diseases such as Alzheimer’s disease (AD) (Fig 5C). These results predict that alterations in HspB2 function may contribute to these diseases.

**Fig 6:**
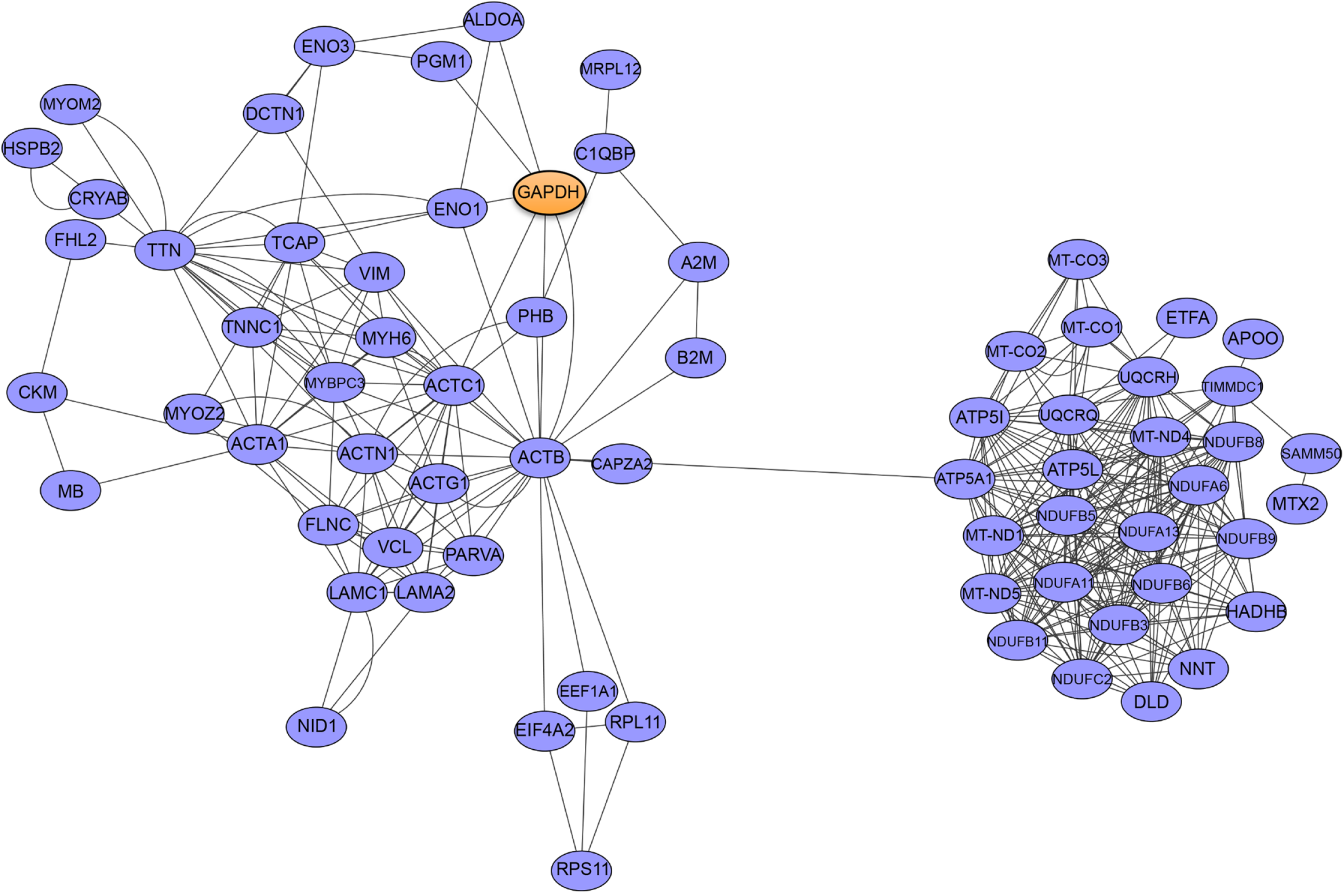
Analysis of the HspB2 interactome confirms the related nature of the binding partners and identified two major networks involved in mitochondrial metabolism and myofibril maintenance. Analysis of known protein-protein interactions among the 149 HspB2 binding partners reveals a tight network of 69 proteins. Glyceraldehyde 3-phosphate dehydrogenase (GAPDH) (indicated by orange coloring) is investigated further in this study. Cytoscape version 3.2.1 [19] was used to construct each of the protein-protein interaction networks by retrieving previously identified interactions from the *mentha*, Reactome-Fls, Reactome, IntAct and MINT databases (partners reported for either human or mouse proteins) and mapping them into a single merged network.

Although the high confidence Y2H and co-IP HspB2 interactome revealed proteins with a wide range of biological functions, it was apparent that many of these proteins overlap in their biological pathways. In order to identify key pathways influenced by HspB2, the combined Y2H and co-IP interactome (149 proteins) was analyzed using protein-protein interaction and functional annotation datasets in Cytoscape [42]. Detailed analysis of the interactome using Cytoscape revealed a large network with 69 of the interactome proteins having 365 reported protein-protein interactions (Fig 6). The remaining 80 proteins not included in this larger network have one or zero known binding partners. There are two major clusters of this large network, one that contains primarily myofibril proteins and the other primarily fermentation/respiration proteins, consistent with the functional analysis of these targets (Figs 4C and 5B). Amid the 64 mitochondria-associated protein partners of HspB2 (19 from Y2H, 48 from co-IP and 3 that overlap), 32 (50%) are in this large Cytoscape network, with 200 of the 365 total interactions (55%) reported occurring with these 32 mitochondria-associated proteins. The fact that many (50%) of the mitochondrial proteins from both screens are previously reported binding partners suggests that HspB2 is targeting related proteins or specific pathways.

Gyceraldehyde 3-phosphate dehydrogenase (GAPDH) is a central protein in the network (indicated in orange) with many connections to the major protein cluster with myofibril functions. GAPDH catalyzes the sixth step of glycolysis and is a key metabolic switch for partitioning glucose to TCA for respiration [43], and has recently been implicated in a wide variety of pathways including mitochondrial functions (for recent reviews see [44–48]). The pivotal position of GAPDH within the cluster and its connection to mitochondrial function suggested GAPDH as an important HspB2 target central to these pathways, and it was investigated further.

### HspB2 protects GAPDH from heat inactivation

GAPDH was chosen to validate by further characterization because it was a central node in the bioinformatic analysis of the HspB2 interactome, it is a soluble protein (facilitating its characterization as chaperone target), and it has been associated with all of the HspB2-related cardiac phenotypes including maintaining ATP levels (Fig 3 and [9]), mitochondrial outer membrane potential ([31, 32]) and protection from I/R-induced infarction (Fig 2, Table 1). Specifically, GAPDH has recently shown to maintain cellular ATP levels during mitochondrial crisis [49], and to localize to mitochondria where it regulates respiratory complex I cytochrome C-oxidase [49], mitochondrial outer membrane permeability (MOMP) [50] and mitochondrial-based apoptosis [50, 51] in cardiomyocytes. These roles for GAPDH in MOMP and apoptosis have also been established in other tissues and cell lines [52–55]. In addition, GAPDH enhances aggregation and cytotoxicity in models of Huntington and Amytrophic Lateral Sclerosis [43, 56] as well as Alzheimer’s disease [57–59].

**Fig 7:**
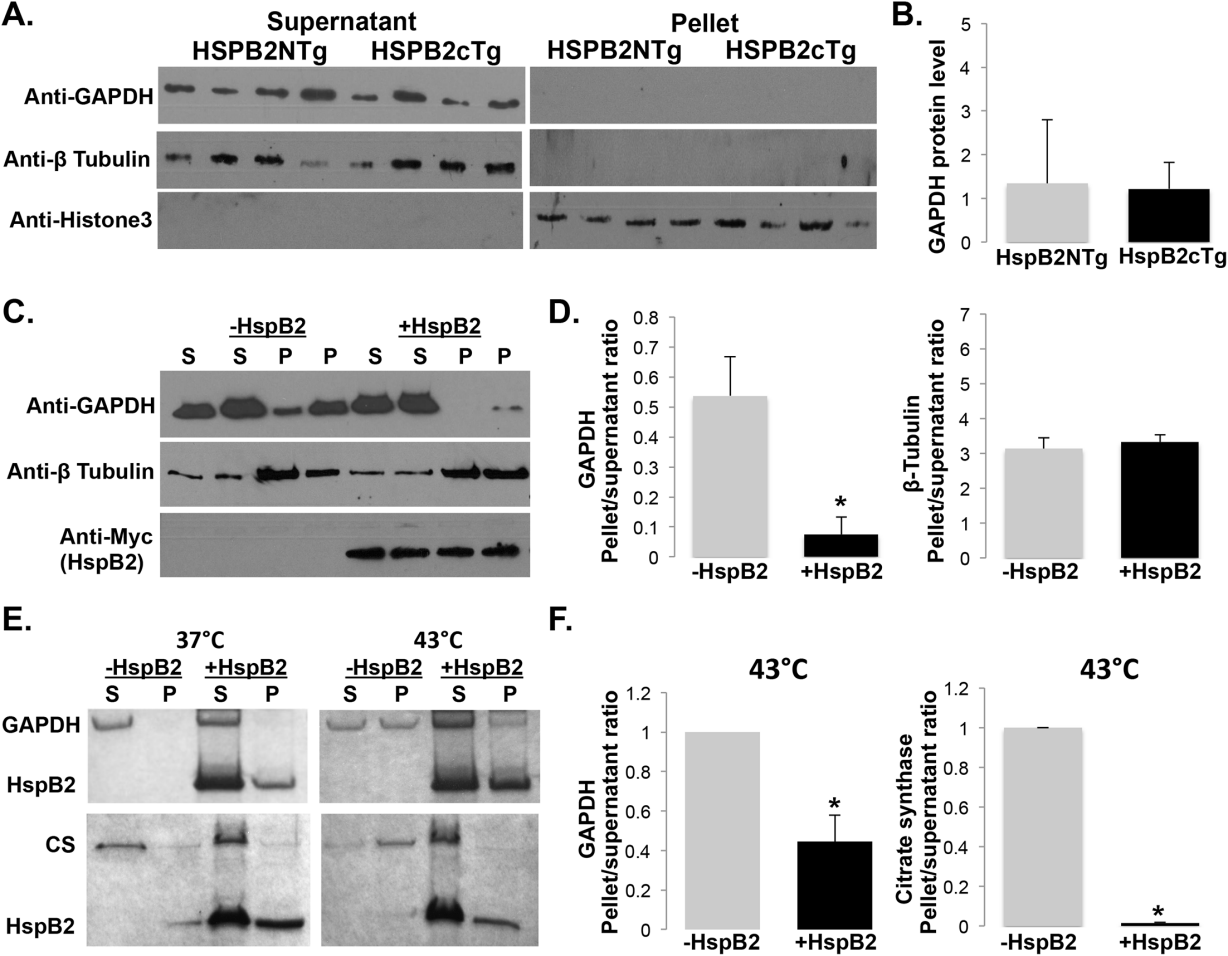
HspB2 protects glyceraldehyde 3-phosphate dehydrogenase (GAPDH) from heat stress. **(A)** Overexpression of HspB2 does not alter GAPDH levels in mouse cardiac lysates. Total protein concentration was determined and western blots were performed using anti-GAPDH with anti-beta Tubulin and anti-histone3 as loading controls as previously described [9]. **(B)** Quantification of A using ImageJ software [22]. **(C)** Overexpression of HspB2 protects GADPH from denaturation in mammalian C2C12 cells subjected to heat shock. Plasmids bearing human HspB2 fused to myc or the empty plasmid control (pCMV-myc) were transfected into C2C12 cells in a 6-well plate using 6.5-μl lipofectamine 2000 (Life Technologies) and manufacturers protocol. After 48 h at 37°C, cells were shifted to 45°C for 30 m and then placed back at 37°C for 3 h prior to lysis. Debris was pelleted at 13,000 rpm for 5 m, and pellet (P) and supernatant (S) were resuspended in 1X SDS buffer. Samples were boiled 2 m, analyzed by SDS-PAGE and transferred to nitrocellulose paper for visualization using anti-GAPDH antibody, anti-beta Tubulin antibody, and anti-Myc antibody (all antibodies are from Cell Signaling Technologies). **(D)** Protein bands from C were quantified using ImageJ software [22]. **(E)** *In vitro* chaperone assay reveals HspB2-dependent protection of GAPDH aggregation, however HspB2 appears to be non-specific in vitro. GAPDH or citrate synthase (control) was incubated with or without HspB2 at 37°C and 43°C and aggregated protein was pelleted by centrifugation. Pellets (P) and supernatants (S) were assayed for protein by SDS-PAGE analysis **(E)** and quantified **(F)**. Briefly, 0.015 mM GAPDH or citrate synthase was incubated at 37°C or 43°C with or without 0.105 mM HspB2 in 40 mM HEPES-KOH buffer (pH 7.5). Aggregates were then pelleted by centrifugation (13,000 rpm for 10 m in a microcentrifuge), run on SDS-PAGE, quantified using ImageJ software [22] and normalized to the no HspB2 pellet to supernatant ratio for each run. Samples were run in triplicate. * *P*<0.05 when compared with corresponding control group.

We tested directly the ability of HspB2 to prevent heat-induced aggregation of GAPDH through *in vivo* and in vitro chaperone assays (Fig 7). To assess the effects of HspB2 on GAPDH *in vivo*, GAPDH expression in cardiac protein lysates from HSPB2cTg mice was compared with the littermate controls (HSPB2NTg). No significant differences in soluble GAPDH were observed when compared with β-Tubulin and GAPDH was not observed in the pellet of homogenized mouse cardiac tissue (Fig 7A and 7B). These results suggest that HspB2 may only be important for GAPDH solubility or folding during stress states. To test this hypothesis, the effects of HspB2 expression on GAPDH heat aggregation were investigated in mammalian cells. In C2C12 cells, HspB2 was overexpressed prior to heat stress (30 m at 45°C) and cell lysates examined for levels of GAPDH contained in the supernatant and pellet fractions. When compared with control cells, forced HspB2 expression significantly prevented aggregation and increased solubilized GAPDH protein (~92% vs 62%, P< 0.005), whereas just over one-third (38%) aggregated, rescuing 30% of the total GAPDH protein (Fig 7C and 7D). In contrast, HspB2 did not recognize β-Tubulin as a binding partner in either the Y2H or co-IP interactomes; accordingly, HspB2 did not confer protection of β-Tubulin when used as a control under these conditions. These results offer for the first time evidence for HspB2-dependent stabilization of GAPDH, a key protein in the regulation of ATP levels and apoptosis.

In addition to *in vivo* assays, the ability of HspB2 to prevent GAPDH aggregation was investigated in vitro. HspB2 was able to prevent *in vitro* aggregation of almost three quarters (~73%) of the GAPDH where ~50% of GAPDH aggregated without HSPB2 (increasing solubility by about half, Fig 7E and 7F). However, HspB2 also prevented aggregation of other proteins tested that were not identified in this study, including citrate synthase (CS). Citrate synthase was reported as a weak client, with HspB2 providing only partial protection in a previous study [21], however these results suggest non-specificity of HspB2 binding *in vitro* with HspB2 rescuing both heat denatured GAPDH and CS.

## Discussion

The physiological importance of the family of sHSPs in human disease has been well established, however the unique roles of the ten members are just beginning to be unveiled due to the complexity of overlapping function [1, 2, 60]. This study utilized the power of OE to reveal cardiac HspB2-associated phenotypes that are not seen with gene KO, most likely due to overlapping sHSP function. In addition, a system’s biology approach was taken to identify specific molecular functions of HspB2 via a cardiac HspB2 interactome, which supports the observed phenotypes and provides key targets for future investigation.

The HspB2 protein was overexpressed in cardiac tissue (HSPB2cTg) where it reduced I/R stress-induced infarct size (Fig 2A and 2B). In support of this finding, the HSPB2cTg animals also had a lower troponin-1 level post surgery (Fig 2C) and were resistant to wall motion abnormality as observed at 24-hour time points post I/R (Table 1). These findings demonstrate the power of OE in the study of complex, overlapping functions, as no difference in infarct size was seen between the wild type (HSPB2wt) and HSPB2cKO mice, which may be due to functional overlap of HSPs. This study of cardiac OE also confirmed the *HSPB2*-dependent preservation of cellular ATP levels seen in the study of HSPB2cKO mice [9], with ATP content following reperfusion significantly greater in the HSPB2cTg mice when compared to the control (Fig 3). These results mimic the function of putative HspB2 activators and suggest them as cardioprotectant agents. In addition, they suggest a mitochondrial role for HspB2 that may explain the protection of cardiac muscle from I/R stress, however a lack of reported mitochondrial HspB2 clientele prohibited molecular analysis.

Complex cellular protein networks underlie most genotype/phenotype relationships, making detailed analysis of protein interactomes essential in functional analysis, particularly with a protein chaperone [61]. Two widely used strategies were employed for determining the HspB2 cardiac interacome; namely, large-scale Y2H screens and co-IP studies. The large-scale cardiac Y2H screen retrieved 84 high confidence *in vivo* binding partners, whereas only four prior HspB2 clientele binding partners were reported to date (DMPK [4], insulin, alcohol dehydrogenase and α-synuclein [21]). Interactions with three other sHSPs (HspB3 [25, 26], HspB5 [27] and HspB8 [28]) have also been previously reported, however, we only retrieved HspB5 from the screen. This may be due to low levels of HspB3 or HspB8 constructs in the Y2H library, or nonfunctional Y2H fusion constructs. The later is highly probable as we were unable to detect an interaction between HspB3 and HspB2 when HspB3 was cloned directly into the Y2H prey (data not shown). The screen would also fail to uncover HspB2 targets that result only from formation of sHSP heterocomplexes (such as an HspB2/HspB5 complex) because only HspB2 was expressed in yeast.

The 84 HspB2 binding partners identified by Y2H are supported by the fact that the HspB2 bait interacted with less than 0.05% of the cardiac cDNA library and was specific for HspB2 when compared to other sHSPs. The HspB2 interactome was compared to the recently published HspB1 (Hsp27) Y2H interactome of 228 binding partners [35], and only two partners were in common (EIF4A2 and GARS). Specificity was also supported by a comparison of HspB2 and HspB5 binding where ten of 12 HspB2 binding partners tested preferentially interacted with HspB2 (see Table 3 and Fig 4A). HspB5 and HspB2, having 43% amino acid identity, are ohnologous [62] proteins in that they are divergently transcribed on human chromosome 11 and appear to have arisen by gene duplication [63]. Differences in function have been attributed to differences in their expression levels and tissue-type patterns, however, the non-redundant clientele-specificity reported herein suggests differential function and supports the unique structure reported for HspB2 when compared with HspB5 [21] as well as the non-redundant cardiac phenotypes [9].

In order to identify key pathways of HspB2 function from among the binding partners, a systems biology approach was used to map previously known protein-protein interactions, protein localization patterns, and related diseases. Such approaches have proven fruitful in identifying key pathways involved in protein function as well as human disease [61]. Functional analysis of the 84 Y2H binding partners revealed an abundance of proteins involved in myofibril and mitochondrial function (Fig 4C). Small HSPs have long been known for stabilizing myofibril structure in both skeletal and cardiac sarcomeres and HspB2 is no exception [6, 36], however this is the first report of putative mitochondrial clientele for HspB2.

The abundance of putative mitochondrial clientele posit HspB2 as a key factor in maintaining cardiac mitochondrial function, particularly when combined with the increased ability to maintain ATP seen in the HSPB2cTg mice (Fig 3). Several of the mitochondrial proteins are reported to localize to the inner membrane and matrix (Table 2) while HspB2 has been associated with the outer mitochondrial membrane, therefore, HspB2 may function as a chaperone for these proteins by helping refold exported, denatured proteins during stress or it may be involved in the import and folding of naïve proteins. In support of a mitochondrial role for HspB2, HspB2 and/or HspB5 have recently been shown to regulate ROS generation and respiration [64]. Cardiac muscle has evolved to be highly resistant to fatigue due to the high proportion of mitochondria within each cardiomyocyte, which is estimated at 40% weight compared with 2% in skeletal muscle [65]. Unlike other muscle, cardiac muscle is almost completely dependent on oxygen with only 1% of energy being derived from anaerobic respiration. Mitochondrial damage is prevalent under growth conditions that stimulate high respiration, making protein chaperones of utmost importance in the heart. We, therefore, set out to validate mitochondrial clientele for HspB2 through co-IP.

The mitochondrial HspB2 clientele obtained by Y2H were supported by 68 proteins retrieved from co-IP with cardiac, mitochondrial HspB2 (Fig 5), with three proteins identified by both methods (HspB2, OGDH, and MCCC2). The low level of overlap between the two independent screens is consistent with previous reports and is due to the differences in whole-cell verses mitochondrial extract as well as the diverse strengths of each approach; co-IP is likely to retrieve large protein complexes while the Y2H primarily identifies direct protein interactions occurring in non-mammalian cells [41]. Although we have only been able to detect HspB2 in mitochondrial fractions when overexpressed in both this study and our previous study [9], HspB2 has also been shown to localize to the mitochondrial membrane in tissue culture studies [8]. This inability to detect HspB2 in mitochondrial fractions under normal cellular conditions has been attributed to its low level of de novo expression as well as its loose association with the mitochondria [9]. The precise role of HspB2 remains to be determined, but the combined phenotypic and interactome results herein strongly argue for a mitochondrial role for HspB2 and identify 64 putative mitochondria-associated clientele to guide future study.

To test the *in vivo* significance of the interactome and analysis, GAPDH was studied in more detail due to its identification as a central hub in the protein-protein interaction network (Fig 6). In addition, GAPDH is soluble, which facilitates chaperone assays and has previously reported roles in each of the HspB2-related phenotypes (namely maintenance of ATP levels and reduced infarct size [49, 50, 52–55]). GAPDH was identified herein as a client through chaperone assays (Fig 7), providing support for the interactome and analysis. Although multiple clienteles are likely to be involved in these phenotypes, GAPDH emerges as a key HspB2 target for further analysis due to its association with mitochondrial ATP production and reduced infarct size.

Although many other sHSPs (including HspB5) have been found to play a role in various diseases including neurodegenerative and muscular disease, disease-associated alleles of HspB2 have not been identified. The systems biology analysis herein, however, posits HspB2 as a player in neurodegenerative, mitochondrial, and muscular disease (Fig 5C). In support of this analysis, a dual KO of *HSPB5* and *HSPB2* was recently shown to enhance Alzheimer’s phenotypes when crossed with AD model mice, including the loss of synapses and the build-up of amyloid beta-peptide (AB) plaques [66]. In addition, HspB2 has recently been found in the senile plaques of AD [67]. This is not surprising given the recently discovered similarities between proteotoxicity in cardiac dysfunction and in AD [68]. Both neurodegenerative and muscular diseases have also been associated with mitochondrial dysfunction [48, 69, 70]. Since the mitochondria of neuronal cells are the main organelle affected during AD [71, 72], mitochondrial dysfunction becomes a primary target for drug development. This analysis of the cardiac HspB2 interactome and cardiac HspB2 OE mice provides insight into the possible effects of treatment with HspB2 activators, including protection from I/R stress and mitochondrial maintenance. The results herein suggest these activators may not only be useful in cardioprotection, but in the treatment of neurodegenerative disease.

## Supporting Information

S1 Fig Mitochondrial permeability assays suggest protection by HspB2 overexpression, however, the data is not statistically significant. Mitochondria were isolated from HSPB2 overexpressors (HSPB2cTg) or control hearts (HSPB2NTg), equilibrated for 5 m at room temperature and 250uM CaCl_2_ was loaded to induce swelling. Data was read at 540nm.
(TIF)

S1 Table Putative HspB2 binding partners retrieved from the Y2H screen only once. Proteins retrieved a single time from a Y2H screen for HspB2 binding partners are given along with the following information: protein name, UniProt [73] protein accession number (hyperlink), gene symbol, and cellular localization reported on UniProt, HPRD or GeneCards [73]. The proteins are functionally categorized by their description on UniProt and/or GeneCards. See Table 2 for a complete list of putative HspB2 binding partners retrieved from the Y2H screen multiple times.
(PDF)
