# Supplementary figures and images for: Characterization of the Cardiac Overexpression of HSPB2 Reveals Mitochondrial and Myogenic Roles Supported by a Cardiac HspB2 Interactome
Source: PLoS One. 2015 Oct 14;10(10):e0133994. doi: 10.1371/journal.pone.0133994 (PMC4605610; doi:10.1371/journal.pone.0133994)

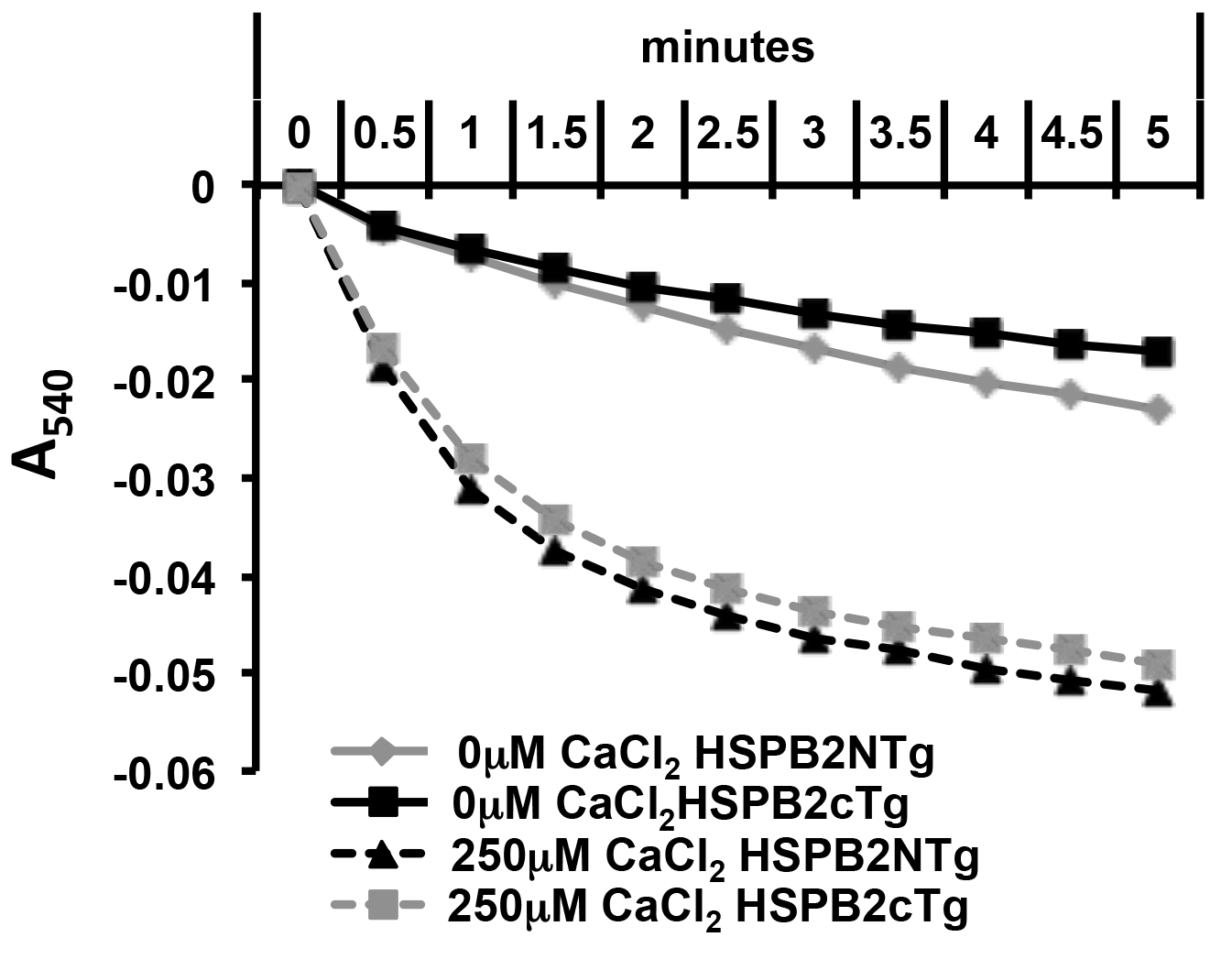

Supplement: S1 Fig — Mitochondria were isolated from HSPB2 overexpressors (HSPB2cTg) or control hearts (HSPB2NTg), equilibrated for 5 m at room temperature and 250uM CaCl2 was loaded to induce swelling. Data was read at 540nm. (TIF) [file pone.0133994.s001.tif]
