# Supplementary material for: Characterization of the Cardiac Overexpression of HSPB2 Reveals Mitochondrial and Myogenic Roles Supported by a Cardiac HspB2 Interactome
Source: PLoS One. 2015 Oct 14;10(10):e0133994. doi: 10.1371/journal.pone.0133994 (PMC4605610; doi:10.1371/journal.pone.0133994)
Supplement: S1 Table — Proteins retrieved a single time from a Y2H screen for HspB2 binding partners are given along with the following information: protein name, UniProt [73] protein accession number (hyperlink), gene symbol, and cellular localization reported on UniProt, HPRD or GeneCards [73]. The proteins are functionally categorized by their description on UniProt and/or GeneCards. See Table 2 for a complete list of putative HspB2 binding partners retrieved from the Y2H screen multiple times. (PDF) [file pone.0133994.s002.pdf]

Supplementary Table 1- Putative HspB2 binding partners retrieved from the Y2H screen only once. Proteins retrieved a single time from a Y2H screen for HspB2 binding partners are given along with the following information: protein name, UniProt [64] protein accession number (hyperlink), gene symbol, and cellular localization reported on UniProt, HPRD or GeneCards [64]. The proteins are functionally categorized by their description on UniProt and/or GeneCards. See Table 2 for a complete list of putative HspB2 binding partners retrieved from the Y2H screen multiple times.

Protein Name	UniProt Accession	Gene Symbol	Cellular Localization**
**Signal Transduction**			
Abhydrolase domain containing 12	Q8N2K0	ABHD12	PM
Active breakpoint cluster region-related protein isoform a	Q12979	ABR	Cytoplasm
ADP-ribosylhydrolase like 2	B7ZAN4	ADPRHL2	Mito
Adrenergic, beta, receptor kinase 1	P25098	ADRBK1	Cytoplasm
Angiotensinogen (serpin peptidase inhibitor, clade A, 8)	P21549	AGT	Extracellular
Adenosylhomocysteinase-like 1	Q9BTL0	AHCYL1	Cytoplasm
A-kinase (PRKA) anchor protein 13	Q12802	AKAP13	PM
Annexin A11	P50995	ANXA11	Cytoplasm
ArfGAP with RhoGAP domain, ankyrin repeat and PH domain 1	Q96P48	ARAP1	Golgi
Calcium/calmodulin-dependent protein kinase II gamma	Q13555	CAMK2G	Cytoplasm
Calcium modulating ligand	P49069	CAMLG	ER
Chordin	Q9H2X0	CHRD	Extracellular
V-crk sarcoma virus CT10 oncogene homolog (avian)-like	P46109	CRKL	Cytoplasm
Dual specificity phosphatase 22	Q9NRW4	DUSP22	Cytoplasm
Epidermal growth factor receptor pathway substrate 8*	Q12929	EPS8	Cytoplasm
Fibroblast growth factor 12 isoform 2	P61328	FGF12	Nucleus
Fms-related tyrosine kinase 1	P17948	FLT1	PM
GRINL1A complex locus	H8Y6P7	GCOM1	PM
Protein-coupled receptor kinase 5	P34947	GRK5	PM
Protein HEG homolog 1	Q9ULI3	HEG1	PM
Latrophilin 1	O94910	LPHN1	PM
Latent-transforming growth factor beta-binding protein 1	Q14766	LTBP1	Extracellular
MAD2L1 binding protein	Q15013	MAD2L1BP	Nucleus
Mitogen activated protein kinase 10	Q02779	MAP3K10	Cytoplasm
Mitogen-inducible gene 6 protein	Q9UJM3	MIG6	Cytoplasm
Nuclear receptor coactivator 4	Q13772	NCOA4	Nucleus
Nischarin	Q9Y2I1	NISCH	PM
NODAL modulator 1	Q15155	NOMO1	ER
NODAL modulator 2 or 3	Q5JPE7<br>P69849	NOMO2(3)	
Nephronectin	Q6UXI9	NPNT	Extracellular
Atrial natriuretic peptide receptor 1 precursor	P16066	NPR1	PM
Optineurin	Q96CV9	OPTN	Nucleus
Platelet-derived growth factor beta polypeptide	P01127	PDGFB	Extracellular
Phosphatidylethanolamine binding protein 1	P30086	PEBP1	Cytoplasm
PTEN induced putative kinase 1	Q9BXM7	PINK1	Mito OM
Pleckstrin homology domain-containing family A member 7	Q6IQ23	PLEKHA7	Cytoplasm
Protein phosphatase 2, catalytic subunit, beta isozyme	P62714	PPP2CB	Cytoplasm
Protein kinase C, beta	P05771	PRKCB	Cytoplasm
Protein kinase, DNA-activated, catalytic polypeptide	P78527	PRKDC	Nucleus
Protein tyrosine phosphatase, non-receptor type 2	P17706	PTPN2	ER

Protein tyrosine phosphatase, receptor type, M	A7MBN1	PTPRM	PM
Rap guanine nucleotide exchange factor (GEF)-like 1	Q9UHV5	RAPGEFL1	PM
Secreted frizzled-related protein 2*	Q96HF1	SFRP2	Extracellular
SMAD family member 4	Q13485	SMAD4	Nucleus
High endothelial venule protein	Q14515	SPARCL1	Extracellular
Serine/threonine kinase 40	Q8N2I9	STK40	Nucleus
Testis expressed 2	Q8IWB9	TEX2	PM
Ubiquitin-like 5	Q9BZL1	UBL5	Cytoplasm
Mitochondrial import stimulation factor L subunit	P62258	YWHAE	Cytoplasm
**Transcription Regulatory Protein**			
Actin-like 6A	O96019	ACTL6A	Nucleus
A-kinase anchor protein 6	Q13023	AKAP6	Nucleus
AT-rich interactive domain-containing protein 4B isoform 1 and 2	Q4LE39	ARID4B	Nucleus
cAMP responsive element binding protein 3	O43889	CREB3	ER
DNA-damage-inducible transcript 3	P35638	DDIT3	Nucleus
E2F transcription factor 6	O75461	E2F6	Nucleus
Histone deacetylase 2	Q92769	HDAC2	Nucleus
Helicase-like transcription factor	Q14527	HLTF	Nucleus
High mobility group nucleosomal binding domain 2	P05204	HMGN2	Nucleus
DNA-binding protein inhibitor ID-3	Q02535	ID3	Nucleus
Integrator complex subunit 4	Q96HW7	INTS4	Nucleus
Integrator complex subunit 10	Q9NVR2	INTS10	Nucleus
KAT8 regulatory NSL complex subunit 3	Q9P2N6	KANSL3	Nucleus
RNA polymerase-associated protein LEO1	Q8WVC0	LEO1	Nucleus
Mbt domain containing 1	Q05BQ5	MBTD1	Nucleus
Nuclear Factor I/A	Q12857	NFIA	Nucleus
Nuclear factor I/X (CCAAT-binding transcription factor)	Q14938	NFIX	Nucleus
Period circadian protein homolog 3	P56645	PER3	Nucleus
PHD finger protein 3	Q92576	PHF3	Nucleus
Polymerase (RNA) II (DNA directed) polypeptide C*	P19387	POLR2C	Nucleus
Polymerase (RNA) II (DNA-directed) polypeptide E	P19388	POLR2E	Nucleus
Peroxisome proliferator-activated receptor alpha	Q07869	PPARA	Nucleus
Peroxisome proliferative activated receptor gamma	P37231	PPARG	Nucleus
RNA binding motif, single stranded interacting protein 1	P29558	RBMS1	Nucleus
Ring finger protein 10	Q8N5U6	RNF10	Cytoplasm
RuvB-like 2	Q9Y230	RUVBL2	Nucleus
SET domain containing (lysine methyltransferase) 7	Q8WTS6	SETD7	Nucleus
Staphylococcal nuclease domain containing 1	Q7KZF4	SND1	Nucleus
Transcription factor 7-like 2 (T-cell specific, HMG-box)	Q9NQB0	TCF7L2	Nucleus
Ubiquitin-conjugating enzyme E2 variant 2	Q15819	UBE2V2	Cytoplasm
Zinc finger and BTB domain containing 16	Q05516	ZBTB16	Nucleus
Zinc finger and BTB domain containing 38	Q8NAP3	ZBTB38	Nucleus
Zinc finger protein 62 homolog	Q8NB50	ZFP62	Nucleus
Zinc finger protein, multitype 2	Q8WW38	ZFPM2	Nucleus
Zinc finger protein 20	Q86XA2	ZNF20	Nucleus
Zinc finger protein 232	Q9UNY5	ZNF232	Nucleus
Zinc finger protein 333	Q96JL9	ZNF333	Nucleus
Zinc finger protein 528	Q3MIS6	ZNF528	Nucleus
Zinc finger family member 673	Q5JUW0	ZNF673	Nucleus
Zinc finger protein 770	Q6IQ21	ZNF770	Nucleus
**Protein Biosynthesis and Processing**			
Caprin cyclase 2	Q6IMN6	CAPRIN2	Cytoplasm, Mito
Cyclin-dependent kinase 12	Q9NYV4	CDK12	Nucleus
DnaJ (Hsp40) homolog, subfamily C, member 24	Q6P3W2	DNAJC24	Cytoplasm
Exoribonuclease family member 3	O43414	ERI3	Nucleus
Heterogeneous nuclear ribonucleoprotein A/B	Q99729	HNRNPAB	Nucleus
Leucyl-tRNA synthetase	Q6NVI4	LARS	Cytoplasm
LSM domain-containing 1	Q9BRA0	LSMD1	Nucleus
Mitochondrial ribosomal protein L16	Q9NX20	MRPL16	Mito
Mitochondrial ribosomal protein L28	Q13084	MRPL28	Mito
Mitochondrial ribosomal protein L48	Q96GC5	MRPL48	Mito
Mitochondrial translation initiation factor 3*	Q9H2K0	MTIF3	Mito
Poly(rC) binding protein 1	Q15365	PCBP1	Mito
Peptidyl-prolyl isomerase E (cyclophilin E)	Q9UNP9	PPIE	Nucleus
Peptidyl-prolyl isomerase F	P30405	PPIF	Mito Matrix
PRKR interacting protein 1	Q9H875	PRKRIP1	Nucleus
Pseudouridylate synthase 3	Q9BZE2	PUS3	Nucleus
KH domain containing, RNA binding	Q96PU8	QKI	Cytoplasm

RNA binding protein fox-1 homolog 1	Q9NWB1	RBFOX1	Nucleus
Ribosomal protein L3	P39023	RPL3	Cytoplasm
Ribosomal protein L7	P18124	RPL7	Ribosome
Ribosomal protein L8	P62917	RPL8	Nucleus
Ribosomal protein L9	Q53Z07	RPL9	Ribosome
Ribosomal protein L10a	P27635	RPL10	Ribosome
Ribosomal protein L12	P30050	RPL12	Nucleus
Ribosomal protein L26	P61254	RPL26	Ribosome
Ribosomal protein L29	P47914	RPL29	PM
Ribosomal protein S8	P62241	RPS8	Cytoplasm
Ribosomal protein S18	P62269	RPS18	Ribosome
Ribosomal protein S27 isoform	P62857	RPS27	Cytoplasm
Ribosomal protein S28	P42677	RPS28	Ribosome
Ribosomal RNA processing protein 1 homolog B	Q14684	RRP1B	Nucleus
Small nuclear ribonucleoprotein polypeptide G	P62308	SNRPG	Nucleus
Serine/arginine-rich splicing factor 3	P84103	SRSF3	Nucleus
Tu translation elongation factor, mitochondrial	P49411	TUFM	Mito
Zinc finger, CCHC domain-containing protein 7	Q8N3Z6	ZCCHC7	Nucleus
**Fermentation/Respiration**			
Aconitase 2	Q99798	ACO2	Mito
Alcohol dehydrogenase 1C (class 1), gamma polypeptide	P00326	ADH1C	Cytoplasm
Cytochrome c oxidase subunit VIa polypeptide 2	Q02221	COX6A2	Mito IM
Cytochrome c oxidase subunit ViiC	P15954	COX7C	Mito IM
Cytochrome c oxidase assembly protein COX11, mitochondrial	Q9Y6N1	COX11	Mito IM
Enoyl CoA hydratase 1, peroxisomal	Q13011	ECH1	Peroxisome
Enoyl-CoA hydratase 3-hydroxyacyl-CoA dehydrogenase	Q08426	EHHADH	Peroxisome
Fission 1 homolog 2	Q9Y3D6	FIS1	Mito OM
Galactose mutarotase (aldose 1-epimerase)	Q96C23	GALM	Cytoplasm
Glutamic-oxaloacetic transaminase 1, soluble	P17174	GOT1	Cytoplasm
Mitochondrial trifunctional enzyme, alpha subunit	P40939	HADHA	Mito IM
Hexokinase 1	P19367	HK1	Cytoplasm
Isocitrate dehydrogenase (NADP)	O75874	IDH1	Cytoplasm
Mannosidase, alpha, class 2B, member 2	O00754	MAN2B1	Lysosome
Mitochondrial encoded cytochrome c oxidase subunit I	P00395	MT-CO1	Mito IM
NADH dehydrogenase (ubiquinone) 1 alpha subcomplex, 3	O95167	NDUFA3	Mito IM
NADH dehydrogenase (ubiquinone) 1 alpha subcomplex 5	Q16718	NDUFA5	Mito IM
NADH dehyrdrogenase (ubiquinone) 1 alpha subcomplex 8*	P51970	NDUFA8	Mito IM
NADH dehydrogenase (ubiquinone) 1 alpha, subcomplex 10	O95299	NDUFA10	Mito IM
NADH dehydrogenase (ubiquinone) 1 beta subcomplex	O95168	NDUFB4	Mito IM
Pyruvate dehydrogenase complex, component X	O00330	PDHX	Mito IM
Phosphoglycerate mutase 2 (muscle)	P15259	PGAM2	Cytoplasm
Phosphoglycerate mutase family member 4	Q8N0Y7	PGAM4	
Succinate-CoA ligase, GDP-forming, beta subunit	Q96I99	SUCLG2	Mito Matrix
Ubiquinol-cytochrome c reductase core protein 1*	P31930	UQCRC1	Mito IM
Ubiquinol-cytochrome c reductase, Rieske iron-sulfur 1	P47985	UQCRFS1	Mito IM
**Myofibril or Cytoskeleton Structure/Regulation**			
Actinin, alpha 1*	P12814	ACTN1	Cytoplasm
ADAM metallopeptidase domain 23*	O75077	ADAM23	PM
Desmin*	P17661	DES	Cytoplasm
Dynein heavy chain domain 1	Q96M86	DNHD1	Cytoskeleton
Adenylyl cyclase-associated protein 1	Q01518	CAP1	Cytoplasm
Calponin 2	Q99439	CNN2	Cytoplasm
Centrobin, centrosomal BRCA2 interacting protein	Q8N137	CNTROB	Cytoskeleton
Gelsolin	P06396	GSN	Extracellular
Kinesin family member 26A	Q9ULI4	KIF26A	Cytoskeleton
LIM domain binding 3	O75112	LDB3	Cytoplasm
Microtubule-associated protein 1B	P46821	MAP1B	Cytoplasm
Marvel domain containing 1	Q9BSK0	MARVELD1	PM
Myosin, heavy chain 9, non-muscle	P35579	MYH9	Cytoplasm
Mysoin 1C*	O00159	MYO1C	PM
Myosin IIIa	Q8NEV4	MYO3A	Cytoplasm
Protein phosphatase 1, regulatory (inhibitor) subunit 12B	Q6GQY8	PPP1R12B	Cytoplasm
RCSD domain containing 1	Q6JBY9	RCSD1	Cytoskeleton
Septin 4	O43236	SEPT4	Cytoplasm,Mito, Nucleus
Sarcoglycan, delta (dystrophin-associated glycoprotein)	Q92629	SGCD	PM
Synemin, intermediate filament protein	O15061	SYNM	Cytoskeleton
Synaptopodin 2	Q9UMS6	SYNPO2	Cytoskeleton
Troponin I type 1 (skeletal, slow)	P19237	TNNI1	Cytoplasm
Utrophin	P46939	UTRN	Cytoplasm

**Small Molecule Biosynthesis and Catabolism**			
4'-phosphopantetheinyl transferase	Q9NRN7	AASDHPPT	Cytoplasm
Acylglycerol kinase	Q53H12	AGK	PM
Glycerol-3-phosphate acyltransferase 3	Q53EU6	AGPAT9	ER
Aldehyde dehydrogenase 1 family, member L1	O75891	ALDH1L1	Cytoplasm
Branched chain amino-acid transaminase 2, mitochondrial	O15382	BCAT2	Mito
CDP-diacylglycerol synthase	O95674	CDS2	Mito IM
Cytochrome P450, family 4, subfamily B, polypeptide 1*	P13584	CYP4B1	ER
7-dehydrocholesterol reductase	Q9UBM7	DHCR7	Peroxisome
Enolase superfamily member 1	Q7L5Y1	ENOSF1	Mito
High density lipoprotein binding protein	Q96CF6	HDLBP	Cytoplasm
Hydroxysteroid (17-beta) dehydrogenase 3	Q5U0Q6	HSD17B3	ER
Iron-sulfur cluster assembly factor homolog	Q5T440	IBA57	Mito Matrix
Ornitine aminotransferase	P04181	OAT	Mito Matrix
Phosphate cytidylyltransferase 2, ethanolamine	Q99447	PCYT2	Cytoplasm
Procollagen-lysine, 2-oxoglutarate 5-dioxygenase 1	Q02809	PLOD1	ER
Phosphopantothenoylcysteine decarboxylase	Q96CD2	PPCDC	Cytoplasm
Retinol saturase (all-trans-retinol 13,14-reductase)	Q6NUM9	RETSAT	ER
Serine hydroxymethyltransferase 2 (mitochondrial)	P34897	SHMT2	Mito IM/Matrix
Trimethyl-lysine hydroxylase, epsilon	Q9NVH6	TMLHE	Mito Matrix
Thiopurine S-methyltransferase	P51580	TPMT	Cytoplasm
**Protein Degradation**			
Cathepsin F	Q9UBX1	CTSF	Lysosome
Cathepsin K	P43235	CTSK	Extracellular
Cullin 5	Q93034	CUL5	Cytoplasm
DDB1 and CUL4 associated factor 8	Q5TAQ9	DCAF8	Nucleus
F-box protein, helicase, 18	Q66K33	FBXO18	Cytoplasm
F-box and WD repeat domain containing 7, E3 ubiquitin ligase	Q969H0	FBXW7	Nucleus
HtrA serine peptidase 1	Q92743	HTRA1	Extracellular
Integral membrane protein 2B	Q9Y287	ITM2B	PM
E3 ubiquitin-protein ligase NEDD4-like	Q96PU5	NEDD4L	PM
Negative regulator of ubiquitin-like proteins 1	Q9Y5A7	NUB1	Nucleus
Pitrilysin metallopeptidase 1	Q5JRX3	PITRM1	Extracellular
Proteasome subunit, beta type, 8*	P28062	PSMB8	Cytoplasm
Ring finger protein 7	Q9UBF6	RNF7	Nucleus
Selenium binding protein 1	Q13228	SELENBP1	Cytoplasm
Ubiquitin A-52 residue ribosomal protein fusion product 1	P62987	UBA52	Ribosome
Ubiquitin C	P0CG48	UBC	Cytoplasm
Ubiquitin ligase E3B	Q7Z3V4	UBE3B	Cytoplasm
Ubiquitin protein ligase E3 component n-recognin 2	Q8IWV8	UBR2	Nucleus
Ubiquitin specific peptidase 9, X-linked	Q86X58	USP9X	Cytoplasm
**Cell Adhesion**			
Multicatalytic endopeptidase complex subunit C13	O75339	PSMB8	Extracellular
Collagen, type I, alpha 2	P08123	COL1A2	Extracellular
Collagen, type XXIII, alpha-1	Q86Y22	COL23A1	PM
Protocadherin 23	Q6V1P9	DCHS2	PM
Desmoplakin*	P15924	DSP	Cytoplasm
Elastin microfibril interfacer 2	Q9BXX0	EMILIN2	Extracellular
Integrin alpha FG-GAP repeat containing 3	Q9H0X4	ITFG3	PM
Integrin, beta 1	P05556	ITGB1	PM
Laminin, alpha 2*	P24043	LAMA2	Extracellular
Nexilin (F actin binding protein)	Q8NBZ9	NEXN	PM
Thrombospondin, type 1, domain-containing 4	Q6ZMP0	THSD4	Extracellular
Tight junction protein 1	Q07157	TJP1	Extracellular
Tensin 1	A1L0S7	TNS1	PM
**Intracellular Trafficking**			
Golgi-associated, gamma adaptin ear containing, ARF binding 1	Q9UJY5	GGA1	Golgi
Golgin A4	Q13439	GOLGA4	Golgi
Gasdermin B	Q8TAX9	GSDMB	Cytoplasm
Insulin-like growth factor 2 receptor	P11717	IGF2R	PM
Lectin, mannose-binding, 1 featuring protein ERGIC-53 precursor	P49257	LMAN1	ER
Lysosomal trafficking regulator	Q99698	LYST	Lysosome
Sorting nexin 3	O60493	SNX3	Cytoplasm
Vesicle-associated membrane protein 3	Q15836	VAMP3	PM
Vesicle-associated membrane protein 7	P51809	VAMP7	Cytoplasm
YKT6 v-SNARE protein	O15498	YKT6	ER
Zinc finger, FYVE domain containing 20	Q9H1K0	ZFYVE20	PM
**Cell Death**			
CLPTM1-like	Q96KA5	CLPTM1L	PM
CASP2 and RIPK1 domain containing adaptor with death domain	P78560	CRADD	Cytoplasm

Defender against cell death 1	P61803	DAD1	ER
Family with sequence similarity 188, member A	Q9H8M7	FAM188A	Nucleus
Hepatitis B virus X interacting protein	O43504	HBXIP	Cytoplasm
LYR motif containing 1	O43325	LYRM1	Nucleus
Melanoma antigen family D, 1*	Q9Y5V3	MAGED1	Cytoplasm
MT-RNR2-like 8	P0CJ75	MTRNR2L8	Cytoplasm
Pleckstring homology-like domain, family A, member 3	Q9Y5J5	PHLDA3	Cytoplasm
Rab interacting lysosomal protein-like	Q5EBL4	RILPL1	Cytoplasm
Transmembrane protein 123	Q8N131	TMEM123	PM
**Transport**			
Aquaporin-7	O14520	AQP7	PM
La ribonucleoprotein domain family, member 1	Q6PKG0	LARP1	Nucleus
Membrane-associated ring finger (C3HC4) 2, E3 ubiquitin protein ligase	Q9P0N8	MARCH2	Lysosome
Solute Carrier Family 41, member 1*	Q8IVJ1	SLC41A1	PM
POM121 membrane glycoprotein	Q9P0N8	POM121	Nucleus
Rab interacting lysosomal protein	Q96NA2	RILP	Lysosome
Solute carrier family 2 (facilitated glucose transporter), member 3	P11169	SLC2A3	PM
Solute carrier family 38, member 1	Q9H2H9	SLC38A1	PM
Phosphodiesterase 4D interacting protein	Q5VU43	PDE4DIP	Golgi
**Osmoregulation**			
ATPase, Na+/K+ transporting, alpha 1 polypeptide	P05023	ATP1A1	PM
ATPase, Ca++ transporting, plasma membrane 4	P23634	ATP2B4	PM
ATP synthase, H+ transporting F1 complex, gamma polypeptide 1	P36542	ATP5C1	Mito IM
ATPase, H+ transporting, lysosomal accessory protein 2	O75787	ATP6AP2	PM
ATPase, class VI, type 11A	P98196	ATP11A	PM
Dicarbonyl/L-xylulose reductase	Q7Z4W1	DCXR	PM
FXYD domain containing ion transport regulator 6	Q9H0Q3	FXYD6	PM
Solute carrier family 9 (sodium, hydrogen exchange), member 1	P19634	SLC9A1	PM
**Immune**			
Armadillo repeat containing, X-linked 1	Q9P291	ARMCX1	PM
Major histocompatibility complex, class 1, A	Q29946	HLA-A	PM
Major histocompatibility complex, class 1, C	Q95HC2	HLA-C	PM
Interferon-related developmental regulator 2	Q12894	IFRD2	Nucleus
Interleukin-1 receptor-associated kinase 1	P51617	IRAK1	Nucleus, Cytoplasm
Lysine (K)-specific dimethylase 5D	Q9BY66	KDM5D	Cytoplasm
Macrophage erythroblast attacher	B4DVN3	MAEA	PM
Thymocyte selection associated family member 2	Q5TEJ8	THEMIS2	Cytoplasm
**Oxidoreductive Stress**			
Glutathione peroxidase 4 (phospholipid hydroperoxidase)	P36969	GPX4	Cytoplasm, Mito IM
Glutathione S-transferase omega 1	P78417	GSTO1	Nucleus
Methionine-R-sulfoxide reductase B1	Q9NZV6	MSRB1	Cytoplasm, Nucleus
Phytanoyl-CoA 2-hydroxylase	O14832	PHYH	Peroxisome
Peroxiredoxin 6	P30041	PRDX6	Lysosome
Selenoprotein W, 1	P63302	SEPW1	Cytoplasm
Serine/threonine kinase 25	O00506	STK25	Golgi
Thioredoxin reductase 1	Q16881	TXNRD1	Cytoplasm
**Nucleotide Metabolism**			
Adenylosuccinate synthase like 1	Q8N142	ADSSL1	Cytoplasm
Adenylate kinase 4	P27144	AK4	Mito Matrix
Branched chain keto acid dehydrogenase E1, alpha polypeptide	P12694	BCKDHA	Mito Matrix
Cytidine deaminase	P32320	CDA	Nucleus
Bis (5'-adenosyl)-triphosphatase	P49789	FHIT	Nucleus
IMP (inosine 5'-monophosphate) dehydrogenase 2	P12268	IMPDH2	Cytoplasm
Nudix (nucleoside diphosphate linked moiety X)-type 7	P0C024	NUDT7	Peroxisome
**Chaperone**			
Ankyrin repeat domain-containing protein 13C	Q8N6S4	ANKRD13C	ER
BCL2-associated athanogene 3	O95817	BAG3	Cytoplasm
Chaperon containing TCP1 subunit 8	Q7Z759	CCT8	Cytoplasm
Cell division cycle 37 homolog (*S. cerevisiae*)	Q16543	CDC37	Cytoplasm
DNAJ (Hsp40) homolog, superfamily B, member 1	P25685	DNAJB1	Cytoplasm
Heat shock protein 90kDA alpha, class A member 1	P07900	HSP90AA1	Cytoplasm
**Iron and Copper Homeostasis**			
Ferritin, heavy polypeptide 1	P02794	FTH1	Cytoplasm
Iron-sulfur cluster assembly enzyme ISCU, mitochondrial*	Q9H1K1	ISCU	Nucleus
Metallothionein 2A*	P02795	MT2A	Nucleus

**Replication/Recombination**			
Kelch domain containing 3	Q8IVW5	KLHDC3	Cytoplasm
Non-structural maintenance of chromosomes element 4, A	Q9NXX6	NSMCE4A	Nucleus
Replication protein A1, 70kDa	P27694	RPA1	Nucleus
**Unknown**			
Aquaporin 7 pseudogene 4		AQP7P4	
Coiled-coil domain containing 130	P13994	CCDC130	Nucleus
Contactin-associated protein-like 3B precursor	Q96NU0	CNTNAP3B	PM
Cytochrome c oxidase assembly factor 6 homolog	Q5JTJ3	COA6	Mito
Beta/gamma crystallin domain-containing protein 3	Q68DQ2	CRYBG3	
Down syndrome critical region gene 6	P57055	DSCR6	Nucleus
ER membrane protein complex subunit 2*	Q15006	EMC2	Cytoplasm
Family with sequence similarity 193, member A	P78312	FAM193A	Nucleus
Family with sequence similarity 214, member A	Q32MH5	FAM214A	
G0/G1 switch 2		GOS2	Golgi
MAP7 domain containing 1	Q3KQU3	MAP7D1	Cytoplasm
Methyltransferase like 2A	Q96IZ6	METTL2A	
Nuclear prelamin A recognition factor	Q9UHQ1	NARF	Nucleus
Protein-L-isoaspartate O-methyltransferase domain containing 1*	Q96MG8	PCMTD1	Cytoplasm
Ribosomal protein L3-like	Q92901	RPL3L	Cytoplasm
Ribosomal protein S2 pseudogene 23		RPS2P23	
RNA pseudouridylate synthase domain containing 2	Q8IZ73	RPUSD2	Cytoplasm
Suppressor of tumorigenicity 7	Q9Y561	ST7	PM
Starch binding domain 1	O95210	STBD1	PM
Tctex1 domain containing 2	Q8WW35	TCTEX1D2	Cytoplasm
Tudor domain containing 10	Q5VZ19	TDRD10	Cytoplasm
Transmembrane protein 143	Q96AN5	TMEM143	Mito
TRAF3IP2 antisense RNA 1 (non-protein coding)		TRAF3IP2-AS1	
Ubiquitin specific peptidase 32 pseudogene 2		USP32P2	
Von Willebrand factor A domain containing 8	A3KMH1	VWA8	Extracellular
YTH domain containing 2	Q9H6S0	YTHDC2	Nucleus
Putative protein kinase regulator			

* Indicates the 20 proteins shown to be HspB2 dependent in the Y2H binding assay. All others were not tested for dependency. Of the proteins tested for dependency, 50% showed a positive Y2H result that was dependent on HspB2 (indicating a HspB2/prey interaction), 12.5% were false positives (they grew with either HspB2 or the empty bait plasmid), and 37.5% were true negatives (they did not grow with either HspB2 or the empty bait). False positives and true negatives revealed in this analysis are not listed.
** Abbreviations include Endoplasmic Reticulum (ER), Mitochondrial (Mito), Mitochondrial Inner Membrane (Mito IM), Mitochondrial Outer Membrane (Mito OM), Mitochondrial Matrix (Mito Matrix), Plasma Membrane (PM)
